# CAR-T Cell Therapy for Breast Cancer: From Basic Research to Clinical Application

**DOI:** 10.7150/ijbs.70120

**Published:** 2022-03-21

**Authors:** Yu-Huan Yang, Jia-Wei Liu, Chen Lu, Ji-Fu Wei

**Affiliations:** 1 Department of Pharmacy, Jiangsu Cancer Hospital, The Affiliated Cancer Hospital of Nanjing Medical University, Jiangsu Institute of Cancer Research, 210009, China.; 2 Department of Clinical Pharmacy, School of Basic Medicine and Clinical Pharmacy, China Pharmaceutical University, Nanjing, 211198, China.; 3 Precision Medicine Center, First Affiliated Hospital of Gannan Medical University, Ganzhou, 341000, China.; 4 Department of Clinical Pharmacy, School of Pharmacy, Nanjing Medical University, Nanjing, 211103, China.

**Keywords:** CAR-T cell therapy, Breast cancer, Preclinical studies, Clinical trials, Tumor-associated antigens

## Abstract

Breast cancer rises as the most commonly diagnosed cancer in 2020. Among women, breast cancer ranks first in both cancer incidence rate and mortality. Treatment resistance developed from the current clinical therapies limits the efficacy of therapeutic outcomes, thus new treatment approaches are urgently needed. Chimeric antigen receptor (CAR) T cell therapy is a type of immunotherapy developed from adoptive T cell transfer, which typically uses patients' own immune cells to combat cancer. CAR-T cells are armed with specific antibodies to recognize antigens in self-tumor cells thus eliciting cytotoxic effects. In recent years, CAR-T cell therapy has achieved remarkable successes in treating hematologic malignancies; however, the therapeutic effects in solid tumors are not up to expectations including breast cancer. This review aims to discuss the development of CAR-T cell therapy in breast cancer from preclinical studies to ongoing clinical trials. Specifically, we summarize tumor-associated antigens in breast cancer, ongoing clinical trials, obstacles interfering with the therapeutic effects of CAR-T cell therapy, and discuss potential strategies to improve treatment efficacy. Overall, we hope our review provides a landscape view of recent progress for CAR-T cell therapy in breast cancer and ignites interest for further research directions.

## Background

The global cancer statistics in 2020 shows breast cancer has overridden lung cancer and become the most commonly diagnosed cancer worldwide, with 2.3 million new cases in 11.7% of all cancer types reported^1^. Moreover, breast cancer mortality ranks the 5^th^, with 685,000 patient deaths in 2020^1^. Among women, breast cancer was diagnosed 1 in 4 cancer cases and 1 in 6 cancer death were caused by breast cancer, ranking the first in both incidence rate and cancer mortality^1^. The histological classification of breast cancer is mainly based on the expression pattern of human epidermal growth factor receptors 2 (HER2) and hormone receptors (HR) named as estrogen receptors (ER) and progesterone receptors (PR), as well as the tumor proliferation rate indicated by Ki-67, resulting in classified 5 major subtypes: HER2 positive, HR positive (HER2^+^ and ER^+^ or PR^+^ or both positive); HER2 positive, HR negative (HER2^+^/ER^-^/PR^-^); basal-like or triple negative (HER2^-^/ER^-^/PR^-^); luminal A (HER2^-^/ER^+^/PR^+^, low proliferation); and luminal B (HER2^-^/ER^+^/PR^+^, high proliferation)^2^. The breast cancer treatment is systemic or local, based on the breast cancer subtype and metastasis degree. For nonmetastatic breast cancer, the main therapeutic goals are eradicating tumors from patients and preventing tumor recurrence. Local therapies including surgery and radiation are used for tumor eradication, while systemic therapies consisting of endocrine therapy, chemotherapy, and immunotherapy are used for further eradication and recurrence prevention. Systemic therapy may be neoadjuvant (preoperative), adjuvant (postoperative), or both. Breast cancer subtypes guide the treatment approaches, such as chemotherapy alone for triple-negative breast cancer, endocrine therapy for all HER2^-^/ER^+^/PR^+^ tumor, and immunotherapy (trastuzumab-based HER2-directed antibody) for all HER2^+^ tumor. For metastatic breast cancer, local therapy approaches along with systemic therapy approaches are typically used to reach the main therapy goals of symptom alleviation and prolonging life^3^.In recent decades, surgery, radiotherapy, chemotherapy, endocrine therapy, targeted therapy, and immunotherapy have improved the survival rate and life quality of breast cancer patients^4^, ^5^. However, the mortality of breast cancer remains high largely due to the fact the developed resistance in patients therapy limits the therapeutic efficacy and treatment outcome^6^-^8^. Therefore, new treatment strategies are urgently needed to further improve breast cancer survival and life quality of patients.

**Figure 1:**
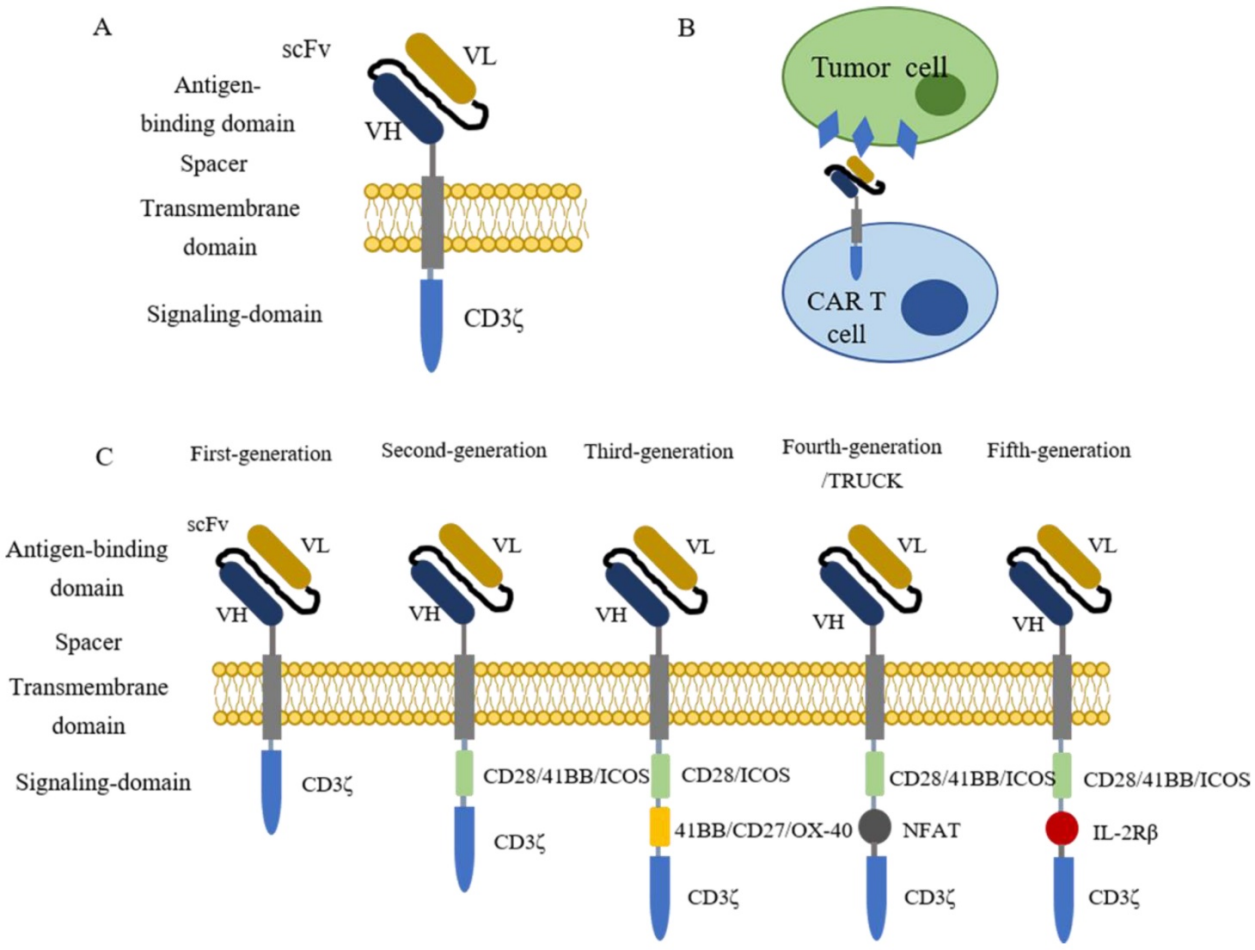
** The introduction of CAR-T cells.** (A) The structure of CARs containing four parts, an extracellular domain containing a single-chain variable fragment (scFv) for antigen recognition, a spacer, a transmembrane domain, and an intracellular signaling domain for T cell activation. (B) CAR-T cell recognizes tumor cells by binding to antigens that present on the surface of tumor cells. (C) The evolution of CAR-T cells. The first generation of CAR-T cells contains one signaling domain (CD3ζ), while two signaling domains (CD3ζ plus CD28/41BB/ICOS) for the second generation. The latter generations contain three intracellular signaling domains. The third generation owns CD3ζ, CD28/ICOS, and 41BB/CD27/OX-40. The fourth and fifth-generation each have a special domain named NFAT and IL-2Rβ plus the normal two signaling domains (CD3ζ and CD28/41BB/ICOS).

Chimeric antigen receptor (CAR) T cell therapy is a type of immunotherapy developed from adoptive T cell transfer (ACT)^9^. In this procedure, patient's T cells are isolated from autologous peripheral blood and further engineered *ex vivo* to express synthetic receptors that recognize tumor-associated antigens (TAAs). Afterwards, CAR-T cells are cultured *ex vivo* for amplification and then infused back to patients as an anti-cancer treatment^10^. Typically, CARs are composed of four segments (Figure 1 A), including an extracellular domain usually containing a single-chain variable fragment (scFv) derived from the variable region of antibodies for tumor antigen recognition, an extracellular spacer regulating the distance between CAR-T cells and tumor cells, a transmembrane domain gluing the synthetic CARs to patient's T cell membrane, and an intracellular signaling domain which consists CD3ζ and costimulatory domains for T cell activation^9^-^14^. When contacting tumor cells, CAR-T cells specifically recognize antigens presenting on the surface of tumor cells (Figure 1B). The clinical results of the first generation of CAR-T cell therapy are unsatisfying because the CAR-T cells show poor persistence and fail for expansion^15^-^17^. To solve these issues, CARs are further engineered with costimulatory signaling domains (Figure 1C). Compared with the first generation of CARs, the second generation adds one costimulatory domain (e.g., CD28, 41BB, ICOS) to the CARs to improve the retention period^18^. The third generation of CARs further include two extra costimulatory domains (e.g., CD27, CD28, 41BB, ICOS, and OX-40) to enhance the persistence and the cytocidal capacity of T cells^19^, ^20^. The fourth generation of CARs, also known as T cells redirected for universal cytokine-mediated killing [TRUCKs], adds a nuclear factor of activated T cells (NFAT) domain harboring an inducible IL-12 cassette^21^, ^22^. In this generation, pro-inflammatory cytokine IL-12 is released and accumulated in the targeted region after CAR-T cells recognize tumor antigens and activate the downstream signaling pathways. Subsequently, the innate immune cells, including NK cells and macrophages, are recruited to tumors to modulate the tumor microenvironment and destroy the cancer cells^22^, ^23^. The fifth generation of CARs is currently under evaluations for safety and efficacy, and it is derived from the second generation of CARs with an extra IL-2 receptor β-chain fragment (IL-2Rβ). The IL-2Rβ fragment bears a binding site to trigger JAK-STAT signaling pathway activation. Once the CAR-T cells target tumor antigens, the antigen-specific activation of the receptor can trigger all the downstream signaling pathways at once, which results in T cells' full activation and persistence enhancement 14, 24, 25.

In 2017, two CAR-T cell therapies targeting CD19 named tisagenlecleucel (Kymriah, Novartis) and axicabtagene ciloleucel (Yescarta, Kite Pharma) were approved by FDA for treating children and young adults with relapsed or refractory acute lymphoblastic leukemia (ALL) and non-Hodgkin's lymphomas (NHLs), respectively^26^-^28^. More recently, another CAR-T cell therapy named as brexucabtagene autoleucel (Tecartus, Kite Pharma) was approved for adult patients with relapsed or refractory mantle cell lymphoma (MCL) in 2020, followed by the approval of Lisocabtagene maraleucel (Breyanzi, BMC) for relapsed or refractory diffuse large B-cell lymphoma (DLBCL) treatment in 2021^29^, ^30^. The successful application of CAR-T cell therapy in hematologic cancers implies that CAR-T cell therapy might be a potential strategy for solid tumors as well. However, unlike hematologic cancers, solid tumors present several barriers to interfere with the activities of CAR-T cells, such as tumor heterogeneity, unfavorable tumor microenvironment, insufficient trafficking and infiltration, and toxicities^11^, ^31^-^33^. Therefore, in recent years, many efforts have been devoted to identifying unique antigens presenting in solid tumor cells and modifying CARs to fit better to solid tumors. Excitingly, several tumor antigens show encouraging results in preclinical studies, including members of receptor tyrosine kinase (RTK)^34^, ^35^, cell surface proteins^36^, ganglioside^37^, stress ligand^38^, tumor serum marker^39^ and others.

In this review, we focus on CAR-T cell therapy in breast cancers. We first summarize tumor antigens in breast cancer and their clinical relevance, followed by the development of antigen-specific CAR-T cells and their applications from basic research to clinical trials. In addition, we also discuss the challenges of CAR-T cell therapy in solid tumors, the efforts developed to overcome these changelings, and the strengths and weaknesses of those approaches. We hope our review will provide a timely update on CAR-T cell therapy in breast cancer and insights to further improve therapeutic efficacy.

## Potential targets for CAR-T cell therapy in breast cancer

**Figure 2:**
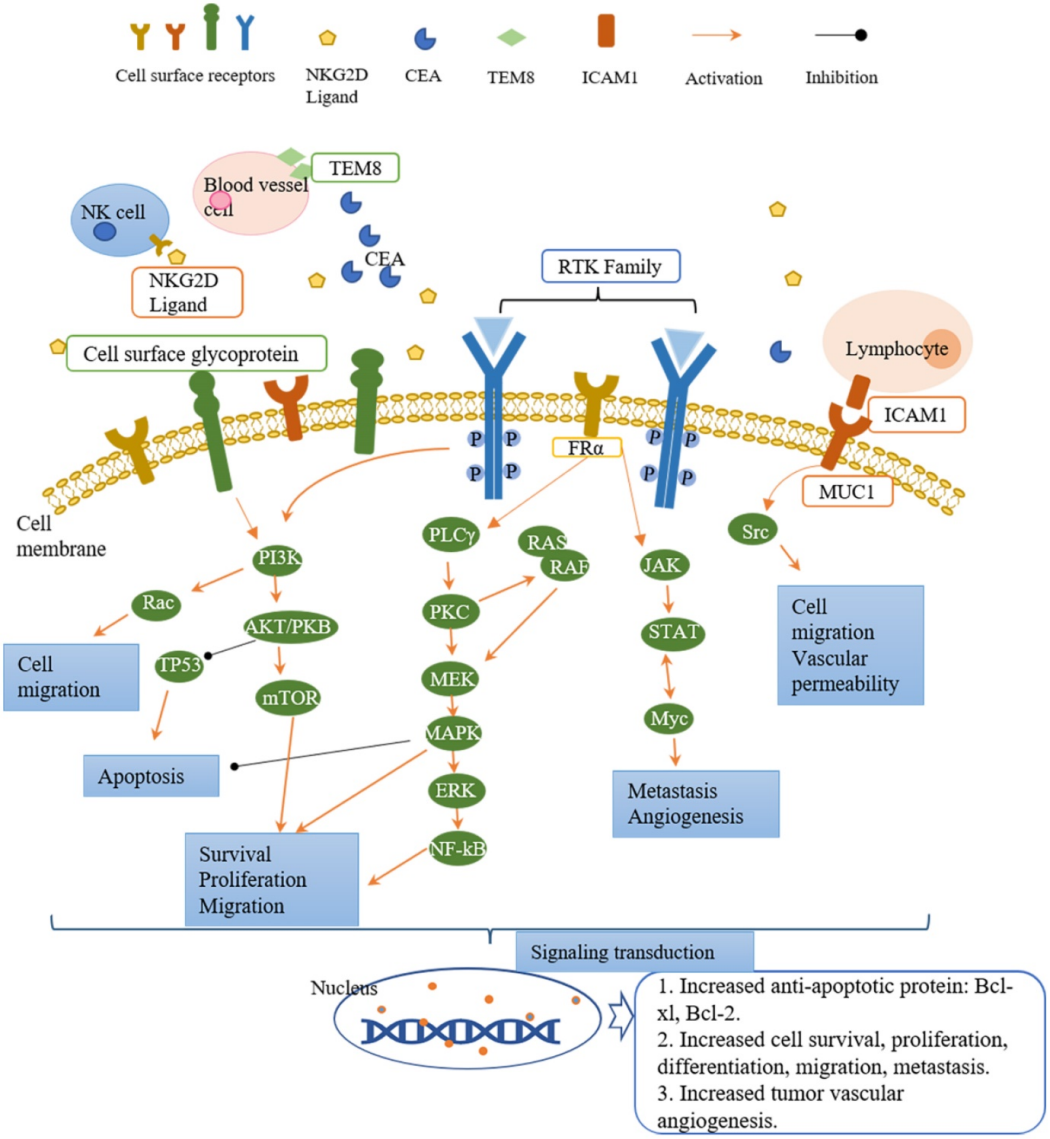
** Targets of CAR-T cell therapy in breast cancer and the downstream signaling pathways involved in cell activity regulation.** The receptors present on the surface of tumor cells, NK cells, lymphocytes, and blood vessel cells. After ligand binding, they activate the downstream signaling pathways to regulate the cell activities. The downstream signaling pathways mainly fall into three cascades, namely PI3K/AKT, PLCγ/PKC, and JAK/STAT. Each cascade contains different downstream molecules. PI3K/AKT pathway and PLCγ/PKC pathway are involved in cell survival, proliferation, migration, and apoptosis. JAK/STAT pathway regulates cell migration and tumor angiogenesis. Down the signaling pathways, the signal is transduced to the nucleus and generates the corresponding proteins (such as anti-apoptotic protein: Bcl-xl, Bcl-2) and activities (such as angiogenesis, cell proliferation, differentiation, migration).

Tumor antigens are divided into three classifications based on the expression pattern, namely tumor-specific antigens (TSAs), tumor-associated antigens (TAAs), and cancer germline antigens (CGAs)^40^. TSAs are the most ideal tumor antigens expressing only on tumor cell surface, including tumor-specific glycosylation (e.g., TnMUC1)^41^. TAAs are more enriched on tumor cells compared with normal tissues (e.g., human epidermal growth factor receptor 2, HER2)^34^, or linear-restrict expression on normal cells (e.g., CD 19)^42^. Targeting TAAs harbors a potential risk for on-target/off-tumor side effects as normal tissues are also attacked by armed CAR-T cells^43^, ^44^. The expression of CGAs is restricted to adult somatic tissues as they are mainly expressed in testis and ovaries^45^. The potential tumor antigens that can be targeted for CAR-T cell therapy in breast cancer are summarized in Table 1. Most of these antigen targets belong to the RTK family and cell surface proteins, while others are stress ligands, disialoganglioside, and serum tumor markers. The downstream signaling pathways of these targets in tumors are summarized in Figure 2. The majority of CAR-T cell targets of breast cancer are TAAs. We will discuss in detail in the following sections for these breast cancer antigens.

### Receptor tyrosine kinase (RTK)

Receptor tyrosine kinases (RTKs) modulate the crucial cell activities such as proliferation, differentiation, metabolism, and survival after activation by growth factors or hormones^35^. Activation of RTKs triggers activation of downstream signaling pathways, including PI3K/AKT, Ras/MEK/ERK, PLCγ/PKC, and JAK/STAT, which plays an essential role in tumor development^34^. PI3K/AKT signaling pathway regulates cell survival, proliferation, migration, and apoptosis. Ras/MEK/ERK and PLCγ/PKC pathways are involved in cell proliferation, migration, and survival, while JAK/STAT pathway modulates angiogenesis and metastasis. In breast cancer, aberrant expression or hyperactivation of several RTKs have been reported including HER2, EGFR^34^. In this section, we focus on five RTKs as CAR-T cell therapy targets.

#### Human epidermal growth factor receptor 2 (HER2)

Human epidermal growth factor receptor 2 (HER2), also known as ERBB2, belongs to the HER/ERBB family of the receptor tyrosine-protein kinase (RTK) family^46^. Once activated, it triggers various downstream signaling pathways to promote expression of genes that encode epithelial-mesenchymal transition (EMT), resulting in the initiation of tumor metastasis^47^-^49^. HER2 dysfunction plays an essential role in the pathogenesis of several tumor types^50^. In breast cancer, nearly 20%-30% of patients are observed with *HER2* gene amplification or HER2 overexpression, correlated with poor clinical outcomes, poor prognosis, and disease progression^51^-^53^. Besides, somatic mutations in *HER2* gene also contribute to breast cancer development through activating HER2 signaling^54^. Therefore, HER2 is a potential target for breast cancer treatments.

The monoclonal antibodies targeting HER2, especially trastuzumab, the first targeted therapy approved by FDA for breast cancer, improved the clinical outcome of breast cancer patients in recent years^55^. In preclinical studies, HER2-CAR-T cells targeting *HER2+* cancers demonstrated significant tumor growth inhibition^56^ and regression of brain tumor metastasis^57^. In addition, in trastuzumab-resistant JIMT-1 cell line derived xenograft mouse models, HER2-CAR-T cells penetrated into the tumor matrix and eradicated the established solid tumor, which subsequently resulted in an improved long-term survival^58^. Besides, even a smaller amount of HER2-targeted CAR-T cells evoked a robust immune reaction and ultimately resulted in tumor remission^59^. Collectively, these results suggest HER2 as a potential target for CAR-T cell therapy in breast cancer.

#### Epidermal growth factor receptor (EGFR)

Epidermal growth factor receptor (EGFR) is also known as ERBB1 or HER1, belonging to the ERBB family. Once activated by ligand binding, it triggers the same downstream signaling pathways as HER2^34^. Approximately 15-30% of breast cancer patients are associated with EGFR overexpression with poor clinical outcomes and larger tumor sizes at diagnosis^60^, ^61^. Notably, EGFR overexpression was mainly observed in triple-negative breast cancer (TNBC), a subtype of breast cancer with estrogen receptor-negative, progestogen receptor-negative, and HER2 negative, accounting for 45-70% of all TNBC patients^62^. Therefore, several EGFR targeted therapies are considered for TNBC treatment, including CAR-T therapy^63^, ^64^. The third-generation of CAR-T cells with an scFv region of anti-EGFR antibodies showed antitumor and cytotoxic effects in TNBC cell cultures (HS578T, MDA-MB-231, MDA-MB-468) and TNBC cell lines derived xenograft mouse models through mechanisms of enhancing cytokine release and tumor lysis^62^.

#### Hepatocyte growth factor receptor (HGFR)

Hepatocyte growth factor receptor (HGFR), also named cMET, belongs to the RTK family and is encoded by a proto-oncogene MET^34^. Once binding to hepatocyte growth factor (HGF), it activates the downstream signaling pathways to regulate tumor progression by controlling the differentiation, proliferation, migration, and apoptosis of tumor cells^65^-^67^. Overexpression of cMET and HGF accounts for 20-30% of breast cancers and is associated with poor prognosis^68^, ^69^. Besides, a study using small-interring RNA to silence the cMET in human TNBC cell lines showed an obvious reduction in tumor cell proliferation and migration^67^. Therefore, targeting cMET might be a potential strategy for breast cancer treatments. Indeed, the TNBC patients receiving cMET-CAR-T cells constructed by mRNA electroporation were well tolerated and the inflammatory response was evoked in tumor sites^70^. More recently, the dual function CAR-T cells targeting cMET and PD-1 at the same tumor cell enhanced the anti-tumor activities and T cell persistence^71^.

#### Receptor tyrosine kinase-like orphan receptor 1 (ROR1)

Receptor tyrosine kinase-like orphan receptor 1(ROR1) also belongs to the RTK family. Highest expression of ROR1 is observed in embryogenesis with reduced expression during fetal development, and eventually disappears in terminally differentiated tissues^72^. Notably, high expression levels of ROR1 is also observed in a few malignancy cancers, including breast cancer^73^, ^74^. In breast cancer, increased ROR1 expression induces expression of ATP-dependent drug efflux pumps (ABCB1), resulting in chemotherapy resistance and tumor recurrence^75^. Notably, chemoresistance could be reversed by using either ROR1 specific antibodies or efflux pump inhibitors^75^, ^76^. Recently, the application of ROR1-CAR-T cells in a 3D microphysiologic tumor models of TNBC presented cytolytic activity and cytokine secretion that favor tumor killing^77^.

#### AXL

AXL is a member of the TAM family of RTKs^78^. Rather than functioning as a driver to initiate cancer transformation, AXL predominantly provides survival, metastatic signals and causes chemo-resistance^79^. Once activated, AXL autophosphorylation stimulates the downstream signaling pathways such as PI3K/AKT, MAPK, and JAK/STAT, therefore, controlling the cancer cell activities^80^. Among all breast cancers, AXL is especially highly expressed in TNBCs hence regarded as a marker of TNBC^81^. Overexpressed AXL is a strong predictor of poor survival and clinical outcomes^82^. The ATP-competitive inhibitors of AXL were reported to limit tumor progression by inhibiting cell activities and inducing apoptosis of breast cancer cells in animal models^83^. Collectively, targeting AXL is a potential strategy for breast cancer treatment. The co-culture of AXL-CAR-T cells and AXL-positive breast cancer cells (such as MDA-MB-231)* in vitro* resulted in increased cytokine release and direct cancer cell lysis compared with AXL-negative cancer cells (such as MCF-7). The same results were observed in MDA-MB-231 derived xenograft mouse models as tumor growth was inhibited by CAR-T therapy^84^. Moreover, the third generation of AXL-CAR-T cells demonstrated anti-tumor effects by inducing cytokine production and cell killing response in AXL-positive cancer cells *in vitro*^85^. In addition, a recent combination strategy of AXL-CAR-T plus constitutive active IL-7 receptor blockade exhibited strong cytotoxic effects *in vitro* and reduced tumor size in MDA-MB-231-derived xenograft mouse models^86^.

### Cell surface proteins

Cell surface proteins are presented on the surface of tumor cells, which acts as tumor antigens for CAR-T cell recognition and aids the antitumor effects of T cells. There are 11 surface proteins with aberrantly increased expression in breast cancer that might be suitable for CAR-T cell therapy.

#### Mucin 1 (MUC1)

Mucin 1 (MUC1), a transmembrane glycoprotein, is usually expressed on the apical side of epithelial cells, secreting mucus to create a chemical barrier to protect host cells from pathogen infection^87^. In the extracellular domain of MUC1, a variable number of tandem repeats (VNTR) region is enriched with serine and proline residues which serves as attachment sites for O-glycans and allows the extensive O-linked glycosylation to take place^88^. The unique enormous O-glycosylation distinguish between tumor and normal cells, rendering MUC1 as an ideal candidate for immunotherapy target^89^. MUC1 overexpression was found in almost 90% of breast cancers^88^. The overexpressed MUC1 regulates tumor migration and progression by decreasing the adhesion capability^90^ and activating the downstream signaling pathways such as ERK1/2 and NFκB^91^. Clinically, breast cancer patients with MUC1 overexpression are associated with poor prognosis and advanced tumor stage^92^.

In TNBC preclinical studies, the second generation of tMUC1-CAR-T cells significantly increased the release of cytokines and chemokines *in vitro* and inhibited tumor growth in 9 TNBC cell lines derived xenograft mouse models^88^. Tn-MUC1 is another form of aberrantly glycated MUC1 expressed in breast cancer^93^. The preclinical studies about TnMUC1-CAR-T cell therapy were less reported, however, a phase I clinical trial of TnMUC1-CAR-T cell therapy was recruiting for TNBC (NCT04025216).

#### Mesothelin (MSLN)

Mesothelin (MSLN) is a cell surface protein and is normally expressed on the surface of mesothelial cells in a few tissues^94^. The biological effects of MSLN remain unclear given that deficiency in *MSLN* gene does not interfere with the development, reproduction, and growth in mouse models^95^. However, overexpression of MSLN has been observed in various solid tumors, including breast cancer^96^, especially in more aggressive and advanced subtypes^97^. Breast cancer patients with MSLN overexpression are correlated with poor clinical outcomes and a greater risk of developing chemo-resistance^98^-^100^. Overexpression of MSLN constitutively activates the intracellular signaling pathways (such as MAPK, PI3K, and NF-kB) to promote tumor development and progression^99^, ^101^. Therefore, targeting MSLN might be a potential strategy for cancer immunotherapies.

The CAR-T cells targeting MSLN in breast cancers mainly focus on TNBC. The third generation of MSLN-CAR-T cells triggered immune responses by releasing cytokines, exhibiting potent cytotoxicity in both MCF-7-Luc and MDA-MB-231-Luc breast cancer cells *in vitro*. Moreover, MSLN-CAR-T cell therapy inhibited tumor growth and metastasis in MDA-MB-231 xenograft models^102^. Programmed cell death protein 1 (PD-1) is an immune checkpoint receptor on the T cell surface, facilitating the cancer cells to escape immune surveillance. The combination of MSLN-CAR-T with programmed cell death protein 1 (PD-1) blockade strongly augmented the cytotoxicity and persistence of T cells within tumors^103^, ^104^. Furthermore, the fourth generation of anti-MSLN-CAR-T cells illustrated a dramatic antitumor capability with complete tumor regression and overall improved survival in mouse models^105^.

#### CD70

CD70 is a costimulatory factor encoded by tumor necrosis factor ligand superfamily member 7 (TNFLS7) and presented on the surface of activated T cells, B cells, and dendritic cells^106^. Normally, CD70 is only expressed in lymphoid tissues^107^, while CD70 overexpression has been found in several solid tumors^108^. Upon binding CD27, CD70 is activated to regulate cell proliferation, survival, and lymphocyte differentiation^109^. The role of CD70 in breast cancer has been controversial^110^. Several immune treatment strategies (such as monoclonal antibodies and CAR-T cells) targeting aberrantly expressed CD70 showed promising results in preclinical and clinical studies. Both humanized anti-CD70 antibodies and anti-CD70 antibody-drug conjugates exhibited remarkable antitumor effects in preclinical researches^109^. In addition, TanCAR-T cells targeting both CD70 and B7-H3 demonstrated enhanced tumor inhibition activities by inducing cytokine release and cytolysis^111^.

#### CD133

CD133, also named prominin 1, is a pentaspan transmembrane glycoprotein normally expressed on the protrusions of the plasma membrane^112^. CD133 has been observed to be enriched in cancer stem cells (CSCs). CSCs are a group of cancer cells sharing stem cell-like features that maintains self-renewal, infinitive differentiation, and proliferation, resulting in high tumorigenicity and treatment resistance^113^, ^114^. CD133 is a biomarker on the surface of CSCs and is regarded as the most rigorous predictor of malignant precursors in various solid tumors, including breast cancer^115^. Besides, CD133 expression is elevated and correlated with poor prognosis and cancer progression in breast cancer patients^112^, ^116^. Collectively, these features suggest CD133 as a potential target for immunotherapy^117^. In fact, CD133-targeted therapies exhibited excellent tumor suppression capacity in several solid tumors. In MDA-MB-231 xenograft models, delivering anti-CD133 antibodies and paclitaxel together significantly improved the therapeutic effects by suppressing the tumor growth and recurrence compared with the paclitaxel only group^118^. However, CD133-CAR-T cells specifically targeting breast cancer studies were less reported. Therefore, more investigations are needed to support the further application of CD133-CAR-T cell therapy in breast cancer.

#### CD44 containing variant exon v6 (CD44v6)

CD44 containing variant exon v6 (CD44v6) is the major variant of CD44, which is a cell surface glycoprotein involved in proliferation, motility, and survival^119^. CD44v6 plays a vital role in tumor development as it activates PI3K/Akt and MAPK signaling pathways to control cell apoptosis, invasion, and metastasis^120^-^122^. Upregulation of CD44v6 has been detected in breast cancers, especially in invasive breast cancer cell lines^123^. Downregulating CD44v6 by microRNA significantly suppressed the invasion and migration of tumor cells^123^. In addition, a meta-analysis showed that the overexpression of CD44v6 was correlated with poor overall survival, breast cancer lymph node metastasis, and more advanced histological stages^124^. These observations suggest CD44v6 as a potential target for breast cancer therapy. Although the preclinical results about CD44v6-CAR-T cell therapy in breast cancer is not well-understood, the clinical trials of anti-CD44v6 CAR-T cells against solid tumors are underway (NCT04427449).

#### Epithelial cell adhesion molecule (EpCAM)

Epithelial cell adhesion molecule (EpCAM) is a glycosylated cell surface protein overexpressed in various epithelial carcinomas, including breast cancer^125^. Highly expressed EpCAM contributes to tumor growth, metastasis, and therapy resistance, leading to shorter disease-free and overall survival in breast cancer patients^126^, ^127^. Therefore, blocking EpCAM on the surface of breast cancer cells can be an approach to inhibit tumor growth, metastasis and improve the therapeutic effects.

Indeed, extensive studies demonstrated the great effects of EpCAM targeted strategies in solid tumor treatments. The antibody-derived EpCAM-targeted methods manifested antitumor effects. For example, adecatumumab targeting EpCAM inhibited breast cancer metastasis in a dose and target-dependent manner, thus catumaxomab targeting EpCAM has already been approved for the treatment of cancers^128^. A cytolytic fusion protein targeting EpCAM illustrated a remarkable tumor inhibiting ability in a human TNBC cell-derived xenograft mouse model^129^. Furthermore, as compared with normal T cells, EpCAM-CAR-T cells induced more cytokine release (such as interferon‑γ, IL‑2, and IL‑6) and exhibited stronger apoptotic effects in cancer cells^130^. Meanwhile, the third generation of EpCAM-CAR-T cells elicited cytotoxic effects in an EpCAM-dependent manner and enormously inhibited tumor formation and growth by secreted high dose of cytokines such as INF-γ and TNF-α in MDA-MB-231 models both *in vitro* and *in vivo*^131^. Taken together, EpCAM as a cancer cell surface antigen displays an important role in targeted therapies.

#### Chondroitin sulfate proteoglycan 4 (CSPG4)

Chondroitin sulfate proteoglycan 4 (CSPG4) is a transmembrane protein overexpressed in several tumors, including breast cancer^132^. Within breast cancer, CSGP4 is highly expressed in more aggressive subtypes, especially basal-like breast cancer and TNBCs^133^, ^134^. Overexpression of CSGP4 plays an essential role in cancer progression and chemoresistance, resulting in poorer overall survival (OS) and shortened recurrence (TTR)^135^. These features make CSGP4 a clinically relevant target for tumor immunotherapy. The anti-CSGP4 monoclonal antibody co-culturing with TNBCs cells inhibited cell migration and growth, and also limited the tumor growth and metastasis in human TNBC cell-derived xenografts in immunodeficient mice by blocking the signaling pathways involved in cell proliferation, migration as well as survival^133^. Furthermore, the second generation of CAR-T cells targeting CSPG4 exhibited cytotoxic effects and induced cytokines production *in vitro*^136^. The CSGP4-CAR-T cells not only inhibited tumor progression by blocking the downstream signaling pathways but also regulated the TME to enhance T cell activities^137^. Together these data suggest that targeting CSGP4 shows an antitumor capability in breast cancer immunotherapies.

#### Intercellular adhesion molecule-1 (ICAM1)

Intercellular adhesion molecule-1 (ICAM1) is a surface glycoprotein that belongs to the immunoglobin superfamily. As a molecular adhesion receptor, ICAM1 plays a role in regulating cell activities including signaling transduction, cell adhesion, and migration^138^, ^139^. Overexpression of ICAM1 has been reported in various cancer, including breast cancer^140^, ^141^. Evidence suggested that increased ICAM1 mRNA and proteins were observed in TNBCs compared with other breast cancer subtypes and normal breast tissues. Blocking ICAM1 by antibodies in highly metastatic MDA-MB-435 cells exhibited a remarkable suppression of cell invasion and migration^141^. Therefore, ICAM1 seems to be a target for therapy. In fact, the coincubation of ICAM1-CAR-T cells with TNBC cells presented the specific and robust killing of cancer cells. While in* in vivo* studies using TNBC cell-derived mouse models, ICAM1-CAR-T cells significantly inhibited tumor growth and resulted in prolonged overall survival and long-term remission^138^.

#### Tumor endothelial marker 8 (TEM8)

Tumor endothelial marker 8 (TEM8), also known as an anthrax toxin receptor 1(ANTXR1), is a group of cell surface glycoproteins. It was identified in terms of its overexpression on epithelial cells of the tumor vasculature and its role in tumor angiogenesis^142^. In breast cancer, elevated expression of TEM8 was associated with a higher risk of tumor relapse^143^. Administration of antibodies blocking TEM8, or genetically knocking out *TEM8* impaired tumor growth and metastasis^142^. The second and third generation of TEM8-specific CAR-T cells targeting TEM8 on TNBC cell lines including MDA-MB-231, MDA-MB-436, MDA-MB-468 and HS578T and human breast tumor endothelial cell line HC6020 demonstrated cytotoxic effects *in vitro* and inhibition of tumor initiation in MDA-MB-468 xenografts^143^. Although targeting TEM8 is a potential strategy to combat breast cancer, further investigations of TEM8-CAR-T cell studies are needed to explore TEM8-specific CARs before clinical studies.

#### Trophoblast cell surface protein 2 (TROP2)

Trophoblast cell surface protein 2 (TROP2) overexpression has been detected in breast cancers, especially TNBCs (approximately 90%)^132^, ^144^. TROP2 is an oncogene that drives cancer cell proliferation, migration, invasion, and metastasis. Overexpression of TROP2 correlates with poor clinical outcomes such as disease progression and short overall survival^145^. Therefore, targeting TROP2 might be a potential approach against TROP2-positive tumors. Indeed, FDA has approved an antibody-drug conjugate called sacituzumab govitecan targeting TROP2 for treating relapsed or refractory metastatic TNBC recently^132^. Human antibody targeting TROP2 elicited antitumor effects both *in vitro* and *in vivo* by inhibiting signaling molecules involved in cell survival^146^. In addition, the TROP2-CAR-T cells based on the CD27 intracellular domain showed stronger antitumor effects than a simple TROP2-CAR-T^145^. Collectively, although targeting TROP2 presents with tumor growth inhibition, the combination with other molecules might provide better clinical outcomes in CAR-T cell therapy.

#### Folate receptor alpha (FRα)

**Figure 3:**
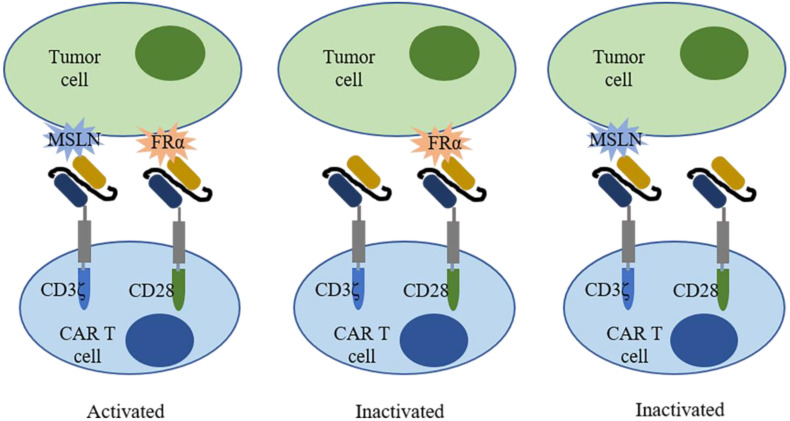
** A trans-signaling CAR-T cell strategy.** A trans-signaling CAR-T cell strategy means T cell activation requires dual recognition and binding between CAR-T cells and antigens. Single recognition of each antigen is not able to activate the CAR-T cell.

Folate receptor alpha (FRα) is a cell surface protein involved in the biosynthesis of nucleotide bases, amino acids, and methylated compounds, playing an important role in cancer development^147^. In breast cancer, overexpression of FRα correlates with poor clinical outcomes such as shorter OS and TTR^148^. Increased expression of FRα on tumor cells renders FRα an attractive target for therapies. In fact, FRα-CAR-T cells effectively targeted FRα-positive TNBC cells and elicited antitumor effects in MDA-MB-231 tumor xenograft, resulting in decreased tumor growth^149^. To improve safety, a trans-signaling CAR strategy was employed^150^. Two different signaling domains (CD3ζ and CD28) were constructed in two separate CARs and in one T cell to target two different antigens (mesothelin and FRα) in one tumor cell (Figure 3). Only when two CARs recognized antigen simultaneously, T cells were activated and elicited antitumor activities. Moreover, a folate-FITC (the conjugation of folate and fluorescein isothiocyanate) acted as a small molecular switch that redirected FITC-CAR-T cells towards FR overexpressing tumor cells also exhibiting cytotoxicity effects^151^.

### Disialoganglioside (GD2)

Disialoganglioside (GD2) is a b-series acidic glycosphingolipid containing two sialic acid residues and facilitates the interaction between tumor cells and extracellular matrix proteins^37^, ^132^. GD2 is a tumor antigen as its expression is highly specific to tumor cells^37^. A small number of cancer cells with GD2 overexpression were able to form mammospheres and initiate tumor formation in mice. However, knockdown of GD3S, an essential enzyme involved in GD2 biosynthesis, completely reversed the tumor formation effects and inhibited tumor metastasis *in vivo*^152^. Moreover, the upregulation of GD2 constitutively activated cMET, a proto-oncogene, resulting in proliferation enhancement, tumor growth, and tumor metastasis^153^, ^154^. These data together suggest GD2 as a candidate for the anticancer target. Dinutuximab targeting GD2 displayed a potent ability of breast cancer suppression in both *in vivo* and* in vitro* studies^155^. It inhibited adhesion of breast cancer cells, migration of breast cancer stem cells, and formation of mammospheres through blocking the mTOR pathway^155^, ^156^. Except for monoclonal antibodies, CAR-T cells targeting GD2 also demonstrated significant antitumor effects in breast cancers^157^. The GD2-CAR-T cells recognized the GD2^+^ breast cancer cells and lysed them *in vitro*, while T cells successfully expanded and trafficked to tumor sites or metastatic tissues to arrest tumor growth and metastasis in MDA-MB-231 derived orthotopic xenograft mouse models^157^. To move GD2-CAR-T cell therapy from basic research to clinical application, clinical trials that test the safety and efficacy of GD2-CAR-T cells are underway.

### Natural killer group 2, member D (NKG2D) Ligand

Natural killer (NK) cells are lymphocytes in the innate immune system, eliminating the target cells and secreting cytokines to establish an adaptive immune response^158^. Natural killer group 2, member D (NKG2D), is a transmembrane glycoprotein on the surface of NK cells and functions as an activating receptor^159^. NKG2D regulates cytotoxicity, cytokine production, and survival^160^, ^161^. NKG2D ligands are highly expressed in the tumor microenvironment with cancer cells, infected cells, and autoimmunity cells^158^. In breast cancer patient samples, evaluation of NKG2D ligand expression showed that it was only expressed on the tumor cells^162^. Undoubtedly, the NKG2D ligand could be a target for cancer immunotherapy. An *in vitro* study using miRNA silencing NKG2D ligand showed that decreased NKG2D ligand improved the NK cell-mediated cytotoxicity and avoided immune escape of breast cancer cells^38^. Moreover, the second-generation of NGK2D-CAR-T cells with the costimulatory domain of 4-1BB/CD27 demonstrated longer T cell persistence *in vivo* and stronger antitumor effects in TNBC cell cultures and mouse models^163^. Tumor models with NKG2D-CAR-T cell therapy illustrated promising results in terms of tumor elimination and tumor-free survival^164^, ^165^. Collectively, NKG2D is a unique antitumor target with improved clinical outcomes in breast cancer.

### Carcinoembryonic antigen (CEA)

Serum tumor markers are a group of proteins that plays an essential role in the early detection of tumor recurrence, metastasis, and screening of many malignancies^166^. Carcinoembryonic antigen (CEA) is one of the most commonly used serum tumor markers in metastatic breast cancer, which normally implies poor overall survival (OS), disease-free survival (DFS), and a higher tumor burden such as lymph node metastasis, advanced TNM stage, and larger tumor size^39^, ^167^. Increased expression of CEA depicts an antitumor probability of CEA-targeting strategies. However, CEA-targeting treatments for breast cancer were less investigated both in preclinical research and clinical studies.

### Other targets

The antigen selection is one of the determining factors of CAR-T cell therapy. In addition to the targets discussed above, other targets have also been explored as potential antigens for CAR-T cell therapy. A *in silico* analysis compared gene expression patterns to identify potential targets for breast cancer immunotherapy, resulting in 36 potentially tumor-surface antigens being discovered, including integrin beta-6 (ITGB6), fibroblast growth factor receptor-4 (FGFR4), and ectonucleotide pyrophosphatase/phosphodiesterase 1 (ENPP1)^168^. Only a few studies focused on those potential targets to clarify their therapeutic effects; therefore, more investigation is needed to estimate their potentiality.

## CAR-T cell therapy clinical trials in breast cancer

Nineteen antigens targeted by CAR-T cells in breast cancer have been well-studied in preclinical studies, resulting in 12 specific antigens progressing into clinical studies for safety and efficacy tests. 22 CAR-constructed T cells targeting 12 antigens have been investigated in recent years (Table 2). We will discuss in detail in the following sections in terms of target type.

### Receptor tyrosine kinase (RTK)

3 of 5 RTK targets have progressed into clinical studies. HER2 is the most frequently used RTK for CAR-T cell therapy in breast cancer as currently there are 3 HER2-CAR-T cell therapy and 1 multi-targets CAR-T cell therapies under clinical trials (NCT04650451, NCT03740256, NCT03696030, and NCT04430595). In 2020, Shanghai PerHum Therapeutics conducted an open-label, single-arm, and dose-escalation phase I clinical study to evaluate the safety, tolerability, and major therapeutic outcomes of CAR-modified autologous T cells in HER2 positive solid tumors including breast cancer (NCT04511871). Besides, there are 2 cMET-CAR-T cell therapy clinical trials granted by the University of Pennsylvania. One was terminated according to funding issues (NCT03060356), while the other one has been completed (NCT01837602) with satisfying clinical results. The results showed that breast cancer patients were well-tolerated to intratumorally injection of cMET-CAR-T cells and none of the patients developed drug-related adverse effects greater than grade 1. In addition, cMET-CAR-T cells elicited antitumor effects by triggering the release of the inflammatory cytokines within the tumor^70^. Furthermore, a phase I clinical trial of ROR1-CAR-T cell therapy was initiated to study the side effects and optimal dose in ROR1-positive cancers including stage IV breast cancers and metastatic TNBC (NCT02706392).

### Cell surface proteins

Although 12 cell surface proteins have been well-studied in preclinical research, only 6 of them progressed into further clinical studies. 3 CAR-T cell therapy clinical trials for mesothelin are currently under investigations in phase-I to test the safety issues (NCT02580747, NCT02792114). In contrast, the rest CARs are undergoing phases-I and II clinical trials to test the safety and efficacy (NCT02414269). Besides, 3 CAR-T cell therapy targeting MUC1 were differently constructed in terms of targets, namely the extra domain of the cleaved form of MUC1 (NCT04020575), the aberrantly glycated MUC1 (NCT04025216), and the whole MUC1 (NCT02587689). Moreover, Shenzhen Geno-Immune Medical Institute starts to recruit individuals for a multicenter phase I/II clinical trial to test the safety and efficacy of CD44v6-specific CAR-engineered T cells in CD44v6 positive cancers (NCT04427449). The 3rd generation of EpCAM-CAR-T cells is ongoing a phase I clinical trial for safety and efficacy determination (NCT02915445). Additionally, a phase I/II clinical trial of CD133-CAR-T cell therapy for investigating safety and T cell duration in patients with relapsed and/or chemotherapy-refractory advanced malignancies was completed recently (NCT02541370) but the results were unrevealed. Apart from that, there was a phase I/II clinical trial of CD70-CAR-T cell therapy for breast cancer patients suspended without reason reported (NCT02830724).

### Other targets

Unlike RKT and cell surface proteins, only a few other targets have entered the stage of clinical trials. 2 CEA-specific CAR-T cell therapies are in phase I clinical trials (NCT02349724, NCT03682744) and 1 in phase I and II clinical trials (NCT04348643). Besides, 2 phase I clinical trials of CAR-T cell therapy targeting NKG2D (NCT04107142) and GD2 (NCT03635632) are ongoing to investigate the safety, efficacy, and tolerability of those treatment strategies.

To sum up, although various CAR-T cell therapy clinical trials have been conducted in recent years, only a few results of those clinical trials are public. To better understand the safety and efficacy of CAR-T cell therapy in breast cancers, the results of those ongoing clinical trials are important, and more clinical trials of CAR-T cells targeting different antigens are warranted.

## CAR-T cell therapy challenges and strategies to overcome these challenges

**Figure 4:**
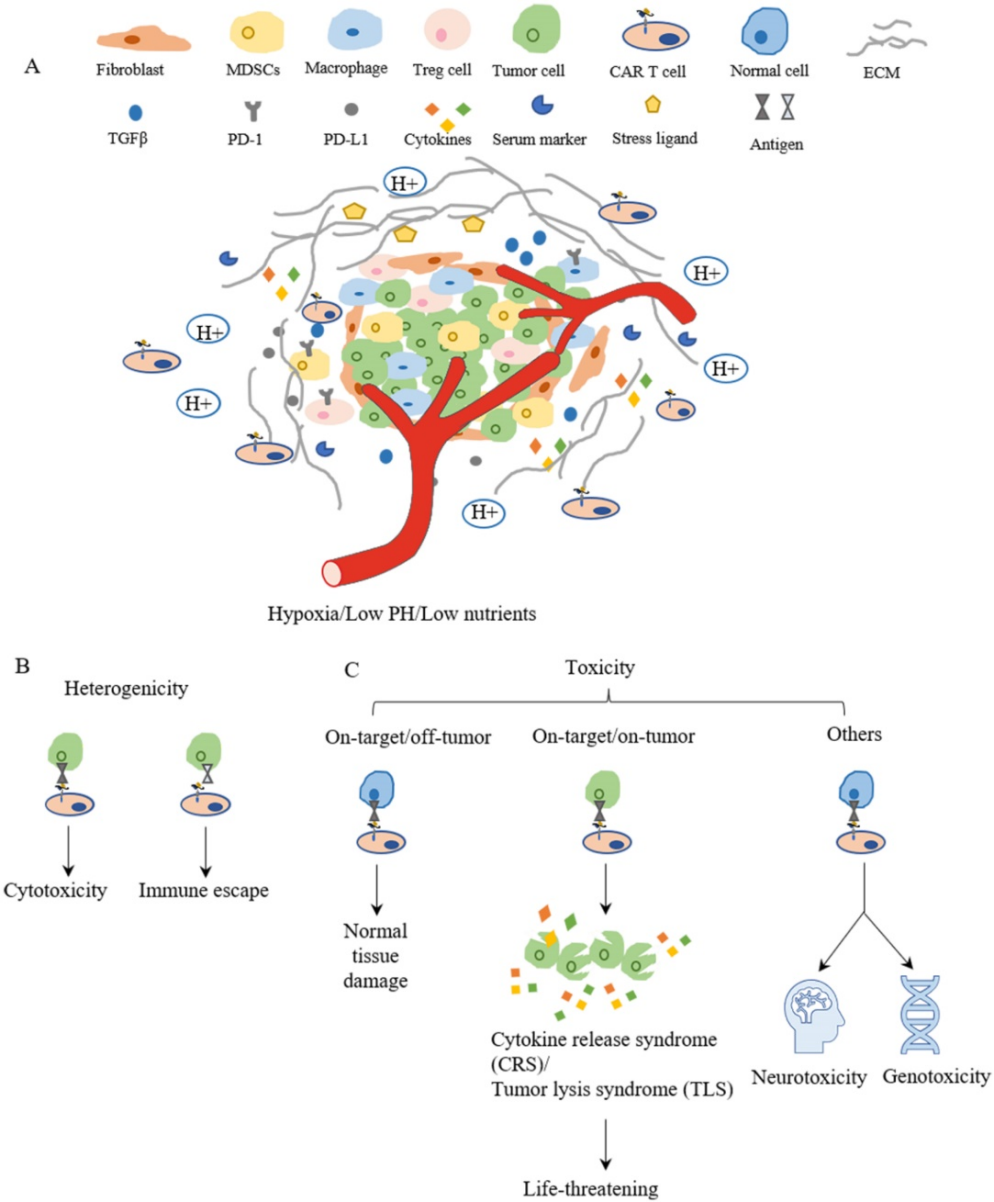
** The main challenges of CAR-T cell therapy.** (A) The tumor microenvironment is lower in oxygen, PH and nutrients, which limits CAR-T cell proliferation and survival. The surrounding fibroblast and ECM inhibit CAR-T cell trafficking and infiltration to tumor sites. The cytokines and checkpoint inhibitors create an immunosuppressive environment around the tumor, which suppresses the function of CAR-T cells. (B) Heterogenicity means different antigen expressions on the surface of tumor cells in terms of type and level, which results in differences in cell response such as cytotoxic or immune escape. (C) The toxicities of CAR-T cell therapy mainly depend on the target. Targeting normal cells results in normal tissue damage or neurotoxicity and genotoxicity. However, life-threatening effects might be evoked when a large amount of tumor cells lysis at the same time and release cytokines and intracellular substances together.

Although CAR-T cell therapies showed some promising results in preclinical studies and in clinical trials for treating breast cancers, challenges remain to restrict the clinical applications and limit therapeutic outcomes. In general, these challenges include insufficient recruiting infiltration of CAR-T cells into tumors, immunosuppressive microenvironment in breast tumors, tumor heterogenicity, and the on-target/off-tumor side effects for CAR-T cells^11^, ^169^ (Figure 4). For each of these major challenges, various strategies have been proposed to further improve the efficacy of CAR-T cell therapy, which are summarized in Table 3.

### Trafficking and infiltration

CAR-T cells must successfully infiltrate into solid tumors such as breast cancer to specifically target tumor cells, which largely depends on the specific binding between chemokine receptors on the surface of CAR-T cells and the chemokines presenting on tumor cells or tumor microenvironment. The chemokines secreted by cancer cells are varied among tumors; hence it is crucial to identify unique chemokine(s) to guide T cells to recognize a given tumor^170^. Unfortunately, the mismatches between chemokine receptors on T cells and chemokines on tumor cells were frequently reported^171^. Two approaches were employed to tackle this problem, which was to design better-matched chemokine receptors on CAR-T cells^172^ and to use oncolytic viruses with chemotactic chemokine to drive CAR-T cells infiltration into tumors^173^. However, it remains challenging to pinpoint the corresponding receptors for chemokines and the loaded viruses might trigger immunogenicity.

In addition, local administration increases the number of CAR-T cells in tumor sites and is suitable for various cancers^169^, ^170^. The limitations associated with this approach are technique challenged than simple intravenous infusion and uneasy to apply on patients with tumors inaccessible by local delivery^11^, ^170^. Moreover, constructing CARs with enzymes to degrade the extracellular matrix of tumor cells or targeting the fibroblast activation protein also aids CAR-T cells infiltration, but the sophisticated modifications might impair T cells' activity^174^, ^175^.

### Tumor immunosuppressive microenvironment

The inhibitory tumor cytokines especially transforming growth factor β (TGFβ) and immunosuppressive cells such as myeloid-derived suppressor cells (MDSCs) and T regulatory cells (Tregs) accumulate together in tumor sites contributing to the immunosuppression of the tumor microenvironment^11^. Overcoming these factors is important for the long-term persistence of T cells and the exhibition of antitumor effects. Inhibiting cytokines by directly constructing T cells with TGFβ receptor or indirectly introducing cytokines (such as IL-2, IL-15, IL-12) to neutralize the immunosuppressive factors improved the T cell persistence and efficacy within tumors. However, T cells eliminated by the host immune system and lack of response towards inhibitory cytokines remain as limitations^176^, ^177^. Furthermore, the combination therapy of CAR-T cells and checkpoint inhibitors particularly anti-PD1 and anti-PD-L1 blockade also demonstrated better therapeutic outcomes than monotherapy, while adding checkpoint inhibitors might increase the probability of immunogenicity^178^.

### Tumor heterogeneity

Tumor heterogeneity means antigen expression variability on the tumor cell surface in terms of type and level, contributing to another layer of challenge for CAR-T cell therapy. Nevertheless, the successful development of multitarget CAR-T cells has partially tackled this obstacle, showing improved antitumor activities in preclinical studies. One of them has moved forward into clinical trials in breast cancer treatment (NCT04430595). In breast cancer, bispecific CAR-T cells targeting HER2 and MUC1 were successfully constructed and exhibited cytotoxic activities^169^. Besides, biCAR, triCAR-T cells also demonstrated antitumor effects^179^, ^180^. The multitarget approach remarkably recruited CAR-T cells in tumor sites and increased the probability of eliminating subpopulation of tumor cells; moreover, it also decreased the risk of on-target/off-tumor side effects to improve safety^181^. However, it is still challenging to select the multi-targets on the same tumor cells and develop the corresponding CAR-T cells.

### CAR-T cell therapy toxicities

Toxicity is one of the major challenges limiting the application of CAR-T cell therapy. The common toxicities are classified into six types, namely on-target on-tumor toxicity, on-target/off-tumor toxicity, off-target toxicity, neurotoxicity, genotoxicity, and immunogenicity^182^. To overcome toxicity issues, plenty of approaches have been developed recently, including multitarget CARs, affinity-tuned CARs, inhibitory CAR (iCARs), introducing suicide genes to CAR-T cells, and using transient RNA expressing CARs^183^. Similar to tumor antigen heterogenicity, CAR-Targeting multi-antigens at once improves safety. Affinity-tuned CARs can distinguish tumor cells and normal cells in terms of antigen expression levels^184^. iCARs are designed to inhibit cytotoxic effects when CAR-T cells target normal cells^185^. However, the effects of these two approaches mainly depend on the expression level of the selected targets. And the inhibitory activity of iCARs might interrupt the antitumor effects of T cells. In addition, incorporating suicide genes such as herpes simplex thymidine kinase (HSV-TK), inducible caspase 9 (iCasp9), and CD20 functioning as safety switches control the cytotoxicity of CAR-T cells and decrease the on-target/off-tumor toxicity^186^, ^187^. Notwithstanding, suicide genes are correlated with some weaknesses, for example, the unintended elimination of the modified functional CAR-T cells, immunogenicity, and long time to affect^188^. Another approach that helps reduce toxicity is to use transient RNA expression of CARs, but it shows insufficient tumor infiltration^170^.

## Conclusion and future perspectives

Breast cancer is currently the most commonly diagnosed cancer around the world, ranking first in both incidence and mortality in women^1^. Resistance development towards the current treatments urges to develop new therapeutics. CAR-T cell therapy is a type of immunotherapies that uses patients' immune cells to fight against cancer^10^. The successful application of CAR-T cell therapy in hematologic malignancies stimulates its expansion in treating solid tumors, including breast cancer. Within breast cancer cells, altered expressions of several molecules are regarded as the potential targets for CAR-T cell therapy.

The present review discusses the development of CAR-T cell therapy from basic research to clinical trials in breast cancer. We reviewed 19 antigens targeted by CAR-T cells in breast cancer, namely HER2, EGFR, HGFR/cMET, ROR1, AXL, MUC1, MSLN, CD70, CD133, CD44v6, EpCAM, CSGP4, ICAM1, TEM8, TROP2, FRα, GD2, NKG2D, CEA, most of which belong to RTK family and cell surface proteins (Table 1). All 19 antigens have been well studied and showed antitumor effects with tumor growth inhibition and proinflammatory cytokines releasing in preclinical studies, whereas only 12 antigens have progressed into clinical trials (Table 2). Although CAR-T cell therapy has made progress in the last few years, it remained several challenges, such as insufficient trafficking and infiltration, the immunosuppressive environment, lack of tumor-specific or tumor-associated antigens, and CAR-T cell toxicities^31^, ^33^. To overcome these challenges and improve CAR-T cell activities, several approaches have been developed for each obstacle (Table 3).

CAR-T cell therapy in breast cancer gains a big achievement till now; however, due to a lack of supporting evidence, it is still a long way from being applied to breast cancer patients. Therefore, to throw CAR-T cell therapy into clinical as early as possible, more clinical trials are needed, and more in-depth investigations are required to improve the safety issues and overcome the challenges of CAR-T cell therapy.

## Figures and Tables

**Table 1:** CAR-T cell targets in breast cancer.

CAR-T cell target	Class of target	Relationship with breast cancer
HER2/ERBB2	Receptor tyrosine kinase (RTK)	Nearly 20%-30% of patients are observed with *HER2* gene amplification or HER2 overexpression, correlated with poor clinical outcomes, poor prognosis, and disease progression^51^-^53^.
EGFR/ERBB1	Receptor tyrosine kinase (RTK)	15-30% of breast cancer patients are associated with EGFR overexpression with poor clinical outcomes and larger tumor sizes at diagnosis^60^, ^61^.
HGFR/cMET	Receptor tyrosine kinase (RTK)	Overexpression of cMET and HGF accounts for 20-30% of breast cancers and is associated with poor prognosis^68^, ^69^.
ROR1	Receptor tyrosine kinase (RTK)	In breast cancer, increased ROR1 expression induces expression of ATP-dependent drug efflux pumps (ABCB1), resulting in chemotherapy resistance and tumor recurrence^75^.
AXL	Receptor tyrosine kinase (RTK)	Overexpressed AXL is a strong predictor of poor survival and clinical outcomes^82^.
MUC1	Cell surface glycoprotein	MUC1 overexpression was found in almost 90% of breast cancers^88^.
MSLN	Cell surface glycoprotein	Breast cancer patients with MSLN overexpression are correlated with poor clinical outcomes and a greater risk of developing chemo-resistance^98^-^100^.
CD70	Cell surface glycoprotein	The role of CD70 in breast cancer has been controversial^110^.
CD133	Cell surface glycoprotein	CD133 expression is elevated and correlated with poor prognosis and cancer progression in breast cancer patients^112^, ^116^.
CD44v6	Cell surface glycoprotein	Upregulation of CD44v6 has been detected in breast cancers, especially in invasive breast cancer cell lines^123^.
EpCAM	Cell surface glycoprotein	Highly expressed EpCAM contributes to tumor growth, metastasis, and therapy resistance, leading to shorter disease-free and overall survival in breast cancer patients^126^, ^127^.
CSGP4	Cell surface glycoprotein	Overexpression of CSGP4 plays an essential role in cancer progression and chemoresistance, resulting in poorer overall survival (OS) and shortened recurrence (TTR)^135^.
ICAM1	Cell surface glycoprotein	Overexpression of ICAM1 has been reported in various cancer, including breast cancer^140^, ^141^.
TEM8	Cell surface glycoprotein	The elevated expression of TEM8 was associated with a higher risk of tumor relapse^143^.
TROP2	Cell surface glycoprotein	Overexpression of TROP2 correlates with poor clinical outcomes such as disease progression and short overall survival^145^.
FRα	Cell surface glycoprotein	In breast cancer, overexpression of FRα correlates with poor clinical outcomes such as shorter OS and TTR^148^.
GD2	Disialoganglioside	GD2 is a tumor antigen as its expression is highly specific to tumor cells^37^.
NGK2D Ligand	Stress ligand	In breast cancer patient samples, evaluation of NKG2D ligand expression showed that it was only expressed on the tumor cells^162^.
CEA	Serum tumor marker	CEA is one of the most commonly used serum tumor markers in metastatic breast cancer, which normally implies poor overall survival, disease-free survival, and a higher tumor burden^39^, ^167^.

**Table 2:** The ongoing clinical trials of CAR-T cell therapy in breast cancer.

ClinicalTrials.number	Status	Estimated enrollement	Targeting antigen	Phase	Indicator	Sponsor
NCT04650451	Recruiting	220	HER2	Phase 1	HER2-positive Breast Cancer	Bellicum Pharmaceuticals
NCT03740256	Recruiting	45	HER2	Phase 1	Breast cancer	Baylor College of Medicine
NCT03696030	Recruiting	39	HER2	Phase 1	Breast Cancer/HER2-positive Breast Cancer	City of Hope Medical Center
NCT04430595	Recruiting	100	Her2-, GD2- and CD44v6	Phase 1,2	Breast Cancer	Shenzhen Geno-Immune Medical Institute
NCT01837602	Completed	6	cMET	Phase 1	Metastatic Breast CancerMetastatic Breast Cancer/Triple Negative Breast Cancer	University of Pennsylvania
NCT03060356	Terminated	77	cMET	Early Phase 1	Breast Cancer	University of Pennsylvania
NCT02706392	Recruiting	60	ROR1	Phase 1	Metastatic Triple-Negative Breast Carcinoma/Stage IV Breast Cancer AJCC v6 and v7	Fred Hutchinson Cancer Research Center
NCT02580747	Unknown	20	Mesothelin	Phase 1	Triple Negative Breast Cancer	Chinese PLA General Hospital
NCT02792114	Recruiting	36	Mesothelin	Phase 1	Breast Cancer/Metastatic HER2-negative Breast	Memorial Sloan Kettering Cancer Center
NCT02414269	Recruiting	179	Mesothelin	Phase 1,2	Breast cancer	Memorial Sloan Kettering Cancer Center
NCT02587689	Unknown	20	MUC1	Phase 1,2	Triple-Negative Invasive Breast Carcinoma	PersonGenBioTherapeutics (Suzhou) Co., Ltd.
NCT04020575	Recruiting	69	MUC1*	Phase 1	Metastatic Breast Cancer	Minerva Biotechnologies Corporation
NCT04025216	Recruiting	112	TnMUC1	Phase 1	Triple Negative Breast Cancer	Tmunity Therapeutics
NCT04427449	Recruiting	100	CD44v6	Phase 1,2	Cancers Which Are CD44v6 Positive	Shenzhen Geno-Immune Medical Institute
NCT02915445	Recruiting	30	EpCAM	Phase 1	Breast Cancer Recurrent	Sichuan University
NCT02541370	Completed	20	CD133	Phase 1,2	Breast cancer	Chinese PLA General Hospital
NCT02830724	Suspension	2	CD70	Phase 1,2	Breast cancer	National Cancer Institute (NCI)
NCT02349724	Unknown	75	CEA	Phase 1	Breast cancer	Southwest Hospital, China
NCT04348643	Recruiting	40	CEA	Phase 1,2	Breast cancer	Chongqing Precision Biotech Co., Ltd
NCT03682744	Active, not recruiting	18	CEA	Phase 1	Breast cancer	Sorrento Therapeutics, Inc.
NCT04107142	Not yet recruiting	10	NKG2D	Phase 1	Triple Negative Breast Cancer	CytoMed Therapeutics Pte Ltd
NCT03635632	Recruiting	94	GD2	Phase 1	Phyllodes Breast Tumor	Baylor College of Medicine

**Table 3:** CAR-T cell therapy Challenges and strategies in breast cancer.

Challenge	Strategies
Trafficking and infiltration	Designing better matched chemokine receptors on CAR-T cell.
	Using oncolytic viruses to drive CAR-T cells traffic to tumor sites.
	Local administration.
	Constructing CARs to degrades extracellular matrix of tumor cells.
	Targeting the fibroblast activation protein.
Tumor immunosuppressive microenvironment	Constructing T cells with TGFβ receptor.
	Introducing cytokines (such as IL-2, IL-15, IL-12) neutralize immunosuppressive factors.
	Combing with checkpoint inhibitors.
Tumor antigen heterogeneity	Developing multitarget CAR-T cells.
Toxicities	Developing multitarget CAR-T cells.
	Developing affinity-tuned CAR-T cells.
	Developing inhibitory CAR-T cells.
	Introducing suicide genes to CAR-T cells.
	Using transient RNA expression of CARs.

## Abbreviations

CAR: chimeric antigen receptor
ACT: adoptive T cell transfer
TAA: tumor-associated antigen
scFv: single-chain variable fragment
TRUCKs: T cells redirected for universal cytokine-mediated killing
NFAT: nuclear factor of activated T cell
NK cell: neutral killer cells
IL-2Rβ: IL-2 receptor β-chain fragment
FDA: food and drug administration
ALL: acute lymphoblastic leukemia
NHL: non-Hodgkin's lymphomas
MCL: mantle cell lymphoma
TME: tumor microenvironment
RTK: receptor tyrosine kinase
TSA: tumor-specific antigens
CGA: cancer germline antigens
HER2: human epidermal receptor 2
EGFR: epidermal growth factor receptor
TNBC: triple negative breast cancer
HGFR: hepatocyte growth factor receptor
HGF: hepatocyte growth factor
ROR1: receptor tyrosine kinase-like orphan receptor 1
ABCB1: ATP-dependent drug efflux pumps
MUC1: mucin 1
VNTR: variable number of tandem repeats
MSLN: mesothelin
PD-1: programmed cell death protein 1
TNFLS7: tumor necrosis factor ligand superfamily member 7
CSC: cancer stem cell
CD44v6: CD44 containing variant exon v6
EpCAM: epithelial cell adhesion molecule
CSPG4: chondroitin sulfate proteoglycan 4
OS: overall survival
TTR: shorter time to recurrence
ICAM1: intercellular adhesion molecule-1
TEM8: tumor endothelial marker 8
ANTXR1: an anthrax toxin receptor 1
BCSC: breast cancer stem cells
TROP2: trophoblast cell surface protein 2
FRα: Folate receptor alpha
GD2: disialoganglioside
NKG2D: natural killer group 2, member D
CEA: carcinoembryonic antigen
DFS: disease-free survival
ITGB6: integrin beta-6
FGFR4: fibroblast growth factor receptor-4
ENPP1: ectonucleotide pyrophosphatase/phosphodiesterase 1
SSEA4: stage-specific embryonic antigen 4
TGFβ: transforming growth factor β
MDSC: myeloid-derived suppressor cells
Tregs: T regulatory cells

## References

[1] Sung H, Ferlay J, Siegel RL, Laversanne M, Soerjomataram I, Jemal A (2021). Global cancer statistics 2020: GLOBOCAN estimates of incidence and mortality worldwide for 36 cancers in 185 countries. CA Cancer J Clin.

[2] Harbeck N, Gnant M (2017). Breast cancer. Lancet.

[3] Waks AG, Winer EP (2019). Breast Cancer Treatment: A Review. Jama.

[4] Figueiredo MI, Cullen J, Hwang YT, Rowland JH, Mandelblatt JS (2004). Breast cancer treatment in older women: does getting what you want improve your long-term body image and mental health?. J Clin Oncol.

[5] Barzaman K, Karami J, Zarei Z, Hosseinzadeh A, Kazemi MH, Moradi-Kalbolandi S (2020). Breast cancer: Biology, biomarkers, and treatments. Int Immunopharmacol.

[6] Palomeras S, Ruiz-Martínez S, Puig T (2018). Targeting Breast Cancer Stem Cells to Overcome Treatment Resistance. Molecules.

[7] DeMichele A, Yee D, Esserman L (2017). Mechanisms of Resistance to Neoadjuvant Chemotherapy in Breast Cancer. N Engl J Med.

[8] Gu G, Dustin D, Fuqua SA (2016). Targeted therapy for breast cancer and molecular mechanisms of resistance to treatment. Curr Opin Pharmacol.

[9] June CH, O'Connor RS, Kawalekar OU, Ghassemi S, Milone MC (2018). CAR T cell immunotherapy for human cancer. Science.

[10] Feins S, Kong W, Williams EF, Milone MC, Fraietta JA (2019). An introduction to chimeric antigen receptor (CAR) T-cell immunotherapy for human cancer. Am J Hematol.

[11] Newick K, O'Brien S, Moon E, Albelda SM (2017). CAR T Cell Therapy for Solid Tumors. Annu Rev Med.

[12] Pang Y, Hou X, Yang C, Liu Y, Jiang G (2018). Advances on chimeric antigen receptor-modified T-cell therapy for oncotherapy. Mol Cancer.

[13] Li S, Siriwon N, Zhang X, Yang S, Jin T, He F (2017). Enhanced Cancer Immunotherapy by Chimeric Antigen Receptor-Modified T Cells Engineered to Secrete Checkpoint Inhibitors. Clin Cancer Res.

[14] Qu J, Mei Q, Chen L, Zhou J (2021). Chimeric antigen receptor (CAR)-T-cell therapy in non-small-cell lung cancer (NSCLC): current status and future perspectives. Cancer Immunol Immunother.

[15] Kershaw MH, Westwood JA, Parker LL, Wang G, Eshhar Z, Mavroukakis SA (2006). A phase I study on adoptive immunotherapy using gene-modified T cells for ovarian cancer. Clin Cancer Res.

[16] Lamers CH, Sleijfer S, Vulto AG, Kruit WH, Kliffen M, Debets R (2006). Treatment of metastatic renal cell carcinoma with autologous T-lymphocytes genetically retargeted against carbonic anhydrase IX: first clinical experience. J Clin Oncol.

[17] Park JR, Digiusto DL, Slovak M, Wright C, Naranjo A, Wagner J (2007). Adoptive transfer of chimeric antigen receptor re-directed cytolytic T lymphocyte clones in patients with neuroblastoma. Mol Ther.

[18] Sadelain M, Brentjens R, Rivière I (2013). The basic principles of chimeric antigen receptor design. Cancer Discov.

[19] D'Aloia MM, Zizzari IG, Sacchetti B, Pierelli L, Alimandi M (2018). CAR-T cells: the long and winding road to solid tumors. Cell Death Dis.

[20] Guedan S, Ruella M, June CH (2019). Emerging Cellular Therapies for Cancer. Annu Rev Immunol.

[21] Chmielewski M, Abken H (2015). TRUCKs: the fourth generation of CARs. Expert Opin Biol Ther.

[22] Chmielewski M, Hombach AA, Abken H (2014). Of CARs and TRUCKs: chimeric antigen receptor (CAR) T cells engineered with an inducible cytokine to modulate the tumor stroma. Immunol Rev.

[23] Chmielewski M, Kopecky C, Hombach AA, Abken H (2011). IL-12 release by engineered T cells expressing chimeric antigen receptors can effectively Muster an antigen-independent macrophage response on tumor cells that have shut down tumor antigen expression. Cancer Res.

[24] Kim DW, Cho JY (2020). Recent Advances in Allogeneic CAR-T Cells. Biomolecules.

[25] Tokarew N, Ogonek J, Endres S, von Bergwelt-Baildon M, Kobold S (2019). Teaching an old dog new tricks: next-generation CAR T cells. Br J Cancer.

[26] Ruella M, Kenderian SS (2017). Next-Generation Chimeric Antigen Receptor T-Cell Therapy: Going off the Shelf. BioDrugs.

[27] Neelapu SS, Locke FL, Bartlett NL, Lekakis LJ, Miklos DB, Jacobson CA (2017). Axicabtagene Ciloleucel CAR T-Cell Therapy in Refractory Large B-Cell Lymphoma. N Engl J Med.

[28] Maude SL, Laetsch TW, Buechner J, Rives S, Boyer M, Bittencourt H (2018). Tisagenlecleucel in Children and Young Adults with B-Cell Lymphoblastic Leukemia. N Engl J Med.

[29] Han D, Xu Z, Zhuang Y, Ye Z, Qian Q (2021). Current Progress in CAR-T Cell Therapy for Hematological Malignancies. J Cancer.

[30] Albinger N, Hartmann J, Ullrich E (2021). Current status and perspective of CAR-T and CAR-NK cell therapy trials in Germany. Gene Ther.

[31] Abreu TR, Fonseca NA, Gonçalves N, Moreira JN (2020). Current challenges and emerging opportunities of CAR-T cell therapies. J Control Release.

[32] Martinez M, Moon EK (2019). CAR T Cells for Solid Tumors: New Strategies for Finding, Infiltrating, and Surviving in the Tumor Microenvironment. Front Immunol.

[33] Knochelmann HM, Smith AS, Dwyer CJ, Wyatt MM, Mehrotra S, Paulos CM (2018). CAR T Cells in Solid Tumors: Blueprints for Building Effective Therapies. Front Immunol.

[34] Hsu JL, Hung MC (2016). The role of HER2, EGFR, and other receptor tyrosine kinases in breast cancer. Cancer Metastasis Rev.

[35] Schlessinger J (2000). Cell signaling by receptor tyrosine kinases. Cell.

[36] Timpe LC, Yen R, Haste NV, Litsakos-Cheung C, Yen TY, Macher BA (2013). Systemic alteration of cell-surface and secreted glycoprotein expression in malignant breast cancer cell lines. Glycobiology.

[37] Ahmed M, Cheung NK (2014). Engineering anti-GD2 monoclonal antibodies for cancer immunotherapy. FEBS Lett.

[38] Shen J, Pan J, Du C, Si W, Yao M, Xu L (2017). Silencing NKG2D ligand-targeting miRNAs enhances natural killer cell-mediated cytotoxicity in breast cancer. Cell Death Dis.

[39] Lee JS, Park S, Park JM, Cho JH, Kim SI, Park BW (2013). Elevated levels of serum tumor markers CA 15-3 and CEA are prognostic factors for diagnosis of metastatic breast cancers. Breast Cancer Res Treat.

[40] Abbott RC, Cross RS, Jenkins MR (2020). Finding the Keys to the CAR: Identifying Novel Target Antigens for T Cell Redirection Immunotherapies. Int J Mol Sci.

[41] Posey AD Jr, Schwab RD, Boesteanu AC, Steentoft C, Mandel U, Engels B (2016). Engineered CAR T Cells Targeting the Cancer-Associated Tn-Glycoform of the Membrane Mucin MUC1 Control Adenocarcinoma. Immunity.

[42] Li X, Ding Y, Zi M, Sun L, Zhang W, Chen S (2017). CD19, from bench to bedside. Immunol Lett.

[43] Morgan RA, Yang JC, Kitano M, Dudley ME, Laurencot CM, Rosenberg SA (2010). Case report of a serious adverse event following the administration of T cells transduced with a chimeric antigen receptor recognizing ERBB2. Mol Ther.

[44] Ahmed N, Brawley VS, Hegde M, Robertson C, Ghazi A, Gerken C (2015). Human Epidermal Growth Factor Receptor 2 (HER2) -Specific Chimeric Antigen Receptor-Modified T Cells for the Immunotherapy of HER2-Positive Sarcoma. J Clin Oncol.

[45] Scanlan MJ, Gure AO, Jungbluth AA, Old LJ, Chen YT (2002). Cancer/testis antigens: an expanding family of targets for cancer immunotherapy. Immunol Rev.

[46] Roskoski R Jr (2014). The ErbB/HER family of protein-tyrosine kinases and cancer. Pharmacol Res.

[47] Chaffer CL, Weinberg RA (2011). A perspective on cancer cell metastasis. Science.

[48] Voutsadakis IA (2019). HER2 in stemness and epithelial-mesenchymal plasticity of breast cancer. Clin Transl Oncol.

[49] Yarden Y, Sliwkowski MX (2001). Untangling the ErbB signalling network. Nat Rev Mol Cell Biol.

[50] Yan M, Schwaederle M, Arguello D, Millis SZ, Gatalica Z, Kurzrock R (2015). HER2 expression status in diverse cancers: review of results from 37,992 patients. Cancer Metastasis Rev.

[51] Borg A, Tandon AK, Sigurdsson H, Clark GM, Fernö M, Fuqua SA (1990). HER-2/neu amplification predicts poor survival in node-positive breast cancer. Cancer Res.

[52] Slamon DJ, Clark GM, Wong SG, Levin WJ, Ullrich A, McGuire WL (1987). Human breast cancer: correlation of relapse and survival with amplification of the HER-2/neu oncogene. Science.

[53] Pegram M, Slamon D (2000). Biological rationale for HER2/neu (c-erbB2) as a target for monoclonal antibody therapy. Semin Oncol.

[54] Comprehensive molecular portraits of human breast tumours Nature. 2012; 490: 61-70.

[55] Carter P, Presta L, Gorman CM, Ridgway JB, Henner D, Wong WL (1992). Humanization of an anti-p185HER2 antibody for human cancer therapy. Proc Natl Acad Sci U S A.

[56] Sun M, Shi H, Liu C, Liu J, Liu X, Sun Y (2014). Construction and evaluation of a novel humanized HER2-specific chimeric receptor. Breast Cancer Res.

[57] Priceman SJ, Tilakawardane D, Jeang B, Aguilar B, Murad JP, Park AK (2018). Regional Delivery of Chimeric Antigen Receptor-Engineered T Cells Effectively Targets HER2(+) Breast Cancer Metastasis to the Brain. Clin Cancer Res.

[58] Szöőr Á, Tóth G, Zsebik B, Szabó V, Eshhar Z, Abken H (2020). Trastuzumab derived HER2-specific CARs for the treatment of trastuzumab-resistant breast cancer: CAR T cells penetrate and eradicate tumors that are not accessible to antibodies. Cancer Lett.

[59] Tóth G, Szöllősi J, Abken H, Vereb G, Szöőr Á (2020). A Small Number of HER2 Redirected CAR T Cells Significantly Improves Immune Response of Adoptively Transferred Mouse Lymphocytes against Human Breast Cancer Xenografts. Int J Mol Sci.

[60] Tsutsui S, Ohno S, Murakami S, Hachitanda Y, Oda S (2002). Prognostic value of epidermal growth factor receptor (EGFR) and its relationship to the estrogen receptor status in 1029 patients with breast cancer. Breast Cancer Res Treat.

[61] Witton CJ, Reeves JR, Going JJ, Cooke TG, Bartlett JM (2003). Expression of the HER1-4 family of receptor tyrosine kinases in breast cancer. J Pathol.

[62] Liu Y, Zhou Y, Huang KH, Li Y, Fang X, An L (2019). EGFR-specific CAR-T cells trigger cell lysis in EGFR-positive TNBC. Aging (Albany NY).

[63] Nakai K, Hung MC, Yamaguchi H (2016). A perspective on anti-EGFR therapies targeting triple-negative breast cancer. Am J Cancer Res.

[64] Tebbutt N, Pedersen MW, Johns TG (2013). Targeting the ERBB family in cancer: couples therapy. Nat Rev Cancer.

[65] Trusolino L, Bertotti A, Comoglio PM (2010). MET signalling: principles and functions in development, organ regeneration and cancer. Nat Rev Mol Cell Biol.

[66] Gaule PB, Crown J, O'Donovan N, Duffy MJ (2014). cMET in triple-negative breast cancer: is it a therapeutic target for this subset of breast cancer patients?. Expert Opin Ther Targets.

[67] Kim YJ, Choi JS, Seo J, Song JY, Lee SE, Kwon MJ (2014). MET is a potential target for use in combination therapy with EGFR inhibition in triple-negative/basal-like breast cancer. Int J Cancer.

[68] Lengyel E, Prechtel D, Resau JH, Gauger K, Welk A, Lindemann K (2005). C-Met overexpression in node-positive breast cancer identifies patients with poor clinical outcome independent of Her2/neu. Int J Cancer.

[69] Ho-Yen CM, Jones JL, Kermorgant S (2015). The clinical and functional significance of c-Met in breast cancer: a review. Breast Cancer Res.

[70] Tchou J, Zhao Y, Levine BL, Zhang PJ, Davis MM, Melenhorst JJ (2017). Safety and Efficacy of Intratumoral Injections of Chimeric Antigen Receptor (CAR) T Cells in Metastatic Breast Cancer. Cancer Immunol Res.

[71] Yuan X, Sun Z, Yuan Q, Hou W, Liang Q, Wang Y (2021). Dual-function chimeric antigen receptor T cells targeting c-Met and PD-1 exhibit potent anti-tumor efficacy in solid tumors. Invest New Drugs.

[72] Fukuda T, Chen L, Endo T, Tang L, Lu D, Castro JE (2008). Antisera induced by infusions of autologous Ad-CD154-leukemia B cells identify ROR1 as an oncofetal antigen and receptor for Wnt5a. Proc Natl Acad Sci U S A.

[73] Balakrishnan A, Goodpaster T, Randolph-Habecker J, Hoffstrom BG, Jalikis FG, Koch LK (2017). Analysis of ROR1 Protein Expression in Human Cancer and Normal Tissues. Clin Cancer Res.

[74] Zhang S, Chen L, Wang-Rodriguez J, Zhang L, Cui B, Frankel W (2012). The onco-embryonic antigen ROR1 is expressed by a variety of human cancers. Am J Pathol.

[75] Fultang N, Illendula A, Lin J, Pandey MK, Klase Z, Peethambaran B (2020). ROR1 regulates chemoresistance in Breast Cancer via modulation of drug efflux pump ABCB1. Sci Rep.

[76] Zhang S, Zhang H, Ghia EM, Huang J, Wu L, Zhang J (2019). Inhibition of chemotherapy resistant breast cancer stem cells by a ROR1 specific antibody. Proc Natl Acad Sci U S A.

[77] Wallstabe L, Göttlich C, Nelke LC, Kühnemundt J, Schwarz T, Nerreter T (2019). ROR1-CAR T cells are effective against lung and breast cancer in advanced microphysiologic 3D tumor models. JCI Insight.

[78] Graham DK, DeRyckere D, Davies KD, Earp HS (2014). The TAM family: phosphatidylserine sensing receptor tyrosine kinases gone awry in cancer. Nat Rev Cancer.

[79] Linger RM, Keating AK, Earp HS, Graham DK (2008). TAM receptor tyrosine kinases: biologic functions, signaling, and potential therapeutic targeting in human cancer. Adv Cancer Res.

[80] Zajac O, Leclere R, Nicolas A, Meseure D, Marchiò C, Vincent-Salomon A (2020). AXL Controls Directed Migration of Mesenchymal Triple-Negative Breast Cancer Cells. Cells.

[81] D'Alfonso TM, Hannah J, Chen Z, Liu Y, Zhou P, Shin SJ (2014). Axl receptor tyrosine kinase expression in breast cancer. J Clin Pathol.

[82] Goyette MA, Duhamel S, Aubert L, Pelletier A, Savage P, Thibault MP (2018). The Receptor Tyrosine Kinase AXL Is Required at Multiple Steps of the Metastatic Cascade during HER2-Positive Breast Cancer Progression. Cell Rep.

[83] Shen Y, Chen X, He J, Liao D, Zu X (2018). Axl inhibitors as novel cancer therapeutic agents. Life Sci.

[84] Wei J, Sun H, Zhang A, Wu X, Li Y, Liu J (2018). A novel AXL chimeric antigen receptor endows T cells with anti-tumor effects against triple negative breast cancers. Cell Immunol.

[85] Cho JH, Okuma A, Al-Rubaye D, Intisar E, Junghans RP, Wong WW (2018). Engineering Axl specific CAR and SynNotch receptor for cancer therapy. Sci Rep.

[86] Zhao Z, Li Y, Liu W, Li X (2020). Engineered IL-7 Receptor Enhances the Therapeutic Effect of AXL-CAR-T Cells on Triple-Negative Breast Cancer. Biomed Res Int.

[87] Nath S, Mukherjee P (2014). MUC1: a multifaceted oncoprotein with a key role in cancer progression. Trends Mol Med.

[88] Zhou R, Yazdanifar M, Roy LD, Whilding LM, Gavrill A, Maher J (2019). CAR T Cells Targeting the Tumor MUC1 Glycoprotein Reduce Triple-Negative Breast Cancer Growth. Front Immunol.

[89] Brockhausen I, Yang JM, Burchell J, Whitehouse C, Taylor-Papadimitriou J (1995). Mechanisms underlying aberrant glycosylation of MUC1 mucin in breast cancer cells. Eur J Biochem.

[90] Suwa T, Hinoda Y, Makiguchi Y, Takahashi T, Itoh F, Adachi M (1998). Increased invasiveness of MUC1 and cDNA-transfected human gastric cancer MKN74 cells. Int J Cancer.

[91] Roy LD, Sahraei M, Subramani DB, Besmer D, Nath S, Tinder TL (2011). MUC1 enhances invasiveness of pancreatic cancer cells by inducing epithelial to mesenchymal transition. Oncogene.

[92] Darlix A, Lamy PJ, Lopez-Crapez E, Braccini AL, Firmin N, Romieu G (2016). Serum HER2 extra-cellular domain, S100ß and CA 15-3 levels are independent prognostic factors in metastatic breast cancer patients. BMC Cancer.

[93] Tarp MA, Sørensen AL, Mandel U, Paulsen H, Burchell J, Taylor-Papadimitriou J (2007). Identification of a novel cancer-specific immunodominant glycopeptide epitope in the MUC1 tandem repeat. Glycobiology.

[94] Ordóñez NG (2003). Application of mesothelin immunostaining in tumor diagnosis. Am J Surg Pathol.

[95] Bera TK, Pastan I (2000). Mesothelin is not required for normal mouse development or reproduction. Mol Cell Biol.

[96] Li YR, Xian RR, Ziober A, Conejo-Garcia J, Perales-Puchalt A, June CH (2014). Mesothelin expression is associated with poor outcomes in breast cancer. Breast Cancer Res Treat.

[97] Klampatsa A, Dimou V, Albelda SM (2021). Mesothelin-targeted CAR-T cell therapy for solid tumors. Expert Opin Biol Ther.

[98] Kachala SS, Bograd AJ, Villena-Vargas J, Suzuki K, Servais EL, Kadota K (2014). Mesothelin overexpression is a marker of tumor aggressiveness and is associated with reduced recurrence-free and overall survival in early-stage lung adenocarcinoma. Clin Cancer Res.

[99] Tozbikian G, Brogi E, Kadota K, Catalano J, Akram M, Patil S (2014). Mesothelin expression in triple negative breast carcinomas correlates significantly with basal-like phenotype, distant metastases and decreased survival. PLoS One.

[100] Tang Z, Qian M, Ho M (2013). The role of mesothelin in tumor progression and targeted therapy. Anticancer Agents Med Chem.

[101] Bharadwaj U, Marin-Muller C, Li M, Chen C, Yao Q (2011). Mesothelin confers pancreatic cancer cell resistance to TNF-α-induced apoptosis through Akt/PI3K/NF-κB activation and IL-6/Mcl-1 overexpression. Mol Cancer.

[102] Li Y, Xiao F, Zhang A, Zhang D, Nie W, Xu T (2020). Oncolytic adenovirus targeting TGF-β enhances anti-tumor responses of mesothelin-targeted chimeric antigen receptor T cell therapy against breast cancer. Cell Immunol.

[103] Hu W, Zi Z, Jin Y, Li G, Shao K, Cai Q (2019). CRISPR/Cas9-mediated PD-1 disruption enhances human mesothelin-targeted CAR T cell effector functions. Cancer Immunol Immunother.

[104] Cherkassky L, Morello A, Villena-Vargas J, Feng Y, Dimitrov DS, Jones DR (2016). Human CAR T cells with cell-intrinsic PD-1 checkpoint blockade resist tumor-mediated inhibition. J Clin Invest.

[105] Adachi K, Kano Y, Nagai T, Okuyama N, Sakoda Y, Tamada K (2018). IL-7 and CCL19 expression in CAR-T cells improves immune cell infiltration and CAR-T cell survival in the tumor. Nat Biotechnol.

[106] Bowman MR, Crimmins MA, Yetz-Aldape J, Kriz R, Kelleher K, Herrmann S (1994). The cloning of CD70 and its identification as the ligand for CD27. J Immunol.

[107] Jilaveanu LB, Sznol J, Aziz SA, Duchen D, Kluger HM, Camp RL (2012). CD70 expression patterns in renal cell carcinoma. Hum Pathol.

[108] Ryan MC, Kostner H, Gordon KA, Duniho S, Sutherland MK, Yu C (2010). Targeting pancreatic and ovarian carcinomas using the auristatin-based anti-CD70 antibody-drug conjugate SGN-75. Br J Cancer.

[109] Grewal IS (2008). CD70 as a therapeutic target in human malignancies. Expert Opin Ther Targets.

[110] Cormary C, Gonzalez R, Faye JC, Favre G, Tilkin-Mariamé AF (2004). Induction of T-cell antitumor immunity and protection against tumor growth by secretion of soluble human CD70 molecules. Cancer Gene Ther.

[111] Yang M, Tang X, Zhang Z, Gu L, Wei H, Zhao S (2020). Tandem CAR-T cells targeting CD70 and B7-H3 exhibit potent preclinical activity against multiple solid tumors. Theranostics.

[112] Li Z, Yin S, Zhang L, Liu W, Chen B, Xing H (2017). Clinicopathological characteristics and prognostic value of cancer stem cell marker CD133 in breast cancer: a meta-analysis. Onco Targets Ther.

[113] Reya T, Morrison SJ, Clarke MF, Weissman IL (2001). Stem cells, cancer, and cancer stem cells. Nature.

[114] Alison MR, Islam S, Wright NA (2010). Stem cells in cancer: instigators and propagators?. J Cell Sci.

[115] Lorico A, Rappa G (2011). Phenotypic heterogeneity of breast cancer stem cells. J Oncol.

[116] Joseph C, Arshad M, Kurozomi S, Althobiti M, Miligy IM, Al-Izzi S (2019). Overexpression of the cancer stem cell marker CD133 confers a poor prognosis in invasive breast cancer. Breast Cancer Res Treat.

[117] Brugnoli F, Grassilli S, Al-Qassab Y, Capitani S, Bertagnolo V (2019). CD133 in Breast Cancer Cells: More than a Stem Cell Marker. J Oncol.

[118] Swaminathan SK, Roger E, Toti U, Niu L, Ohlfest JR, Panyam J (2013). CD133-targeted paclitaxel delivery inhibits local tumor recurrence in a mouse model of breast cancer. J Control Release.

[119] Naor D, Sionov RV, Ish-Shalom D (1997). CD44: structure, function, and association with the malignant process. Adv Cancer Res.

[120] Marhaba R, Bourouba M, Zöller M (2005). CD44v6 promotes proliferation by persisting activation of MAP kinases. Cell Signal.

[121] Jung T, Gross W, Zöller M (2011). CD44v6 coordinates tumor matrix-triggered motility and apoptosis resistance. J Biol Chem.

[122] Günthert U, Hofmann M, Rudy W, Reber S, Zöller M, Haussmann I (1991). A new variant of glycoprotein CD44 confers metastatic potential to rat carcinoma cells. Cell.

[123] Hu S, Cao M, He Y, Zhang G, Liu Y, Du Y (2020). CD44v6 Targeted by miR-193b-5p in the Coding Region Modulates the Migration and Invasion of Breast Cancer Cells. J Cancer.

[124] Qiao GL, Song LN, Deng ZF, Chen Y, Ma LJ (2018). Prognostic value of CD44v6 expression in breast cancer: a meta-analysis. Onco Targets Ther.

[125] Spizzo G, Gastl G, Wolf D, Gunsilius E, Steurer M, Fong D (2003). Correlation of COX-2 and Ep-CAM overexpression in human invasive breast cancer and its impact on survival. Br J Cancer.

[126] Gastl G, Spizzo G, Obrist P, Dünser M, Mikuz G (2000). Ep-CAM overexpression in breast cancer as a predictor of survival. Lancet.

[127] Mal A, Bukhari AB, Singh RK, Kapoor A, Barai A, Deshpande I (2020). EpCAM-Mediated Cellular Plasticity Promotes Radiation Resistance and Metastasis in Breast Cancer. Front Cell Dev Biol.

[128] Eyvazi S, Farajnia S, Dastmalchi S, Kanipour F, Zarredar H, Bandehpour M (2018). Antibody Based EpCAM Targeted Therapy of Cancer, Review and Update. Curr Cancer Drug Targets.

[129] Amoury M, Kolberg K, Pham AT, Hristodorov D, Mladenov R, Di Fiore S (2016). Granzyme B-based cytolytic fusion protein targeting EpCAM specifically kills triple negative breast cancer cells *in vitro* and inhibits tumor growth in a subcutaneous mouse tumor model. Cancer Lett.

[130] Zhou Y, Wen P, Li M, Li Y, Li XA (2019). Construction of chimeric antigen receptor-modified T cells targeting EpCAM and assessment of their anti-tumor effect on cancer cells. Mol Med Rep.

[131] Zhang BL, Li D, Gong YL, Huang Y, Qin DY, Jiang L (2019). Preclinical Evaluation of Chimeric Antigen Receptor-Modified T Cells Specific to Epithelial Cell Adhesion Molecule for Treating Colorectal Cancer. Hum Gene Ther.

[132] Dees S, Ganesan R, Singh S, Grewal IS (2020). Emerging CAR-T Cell Therapy for the Treatment of Triple-Negative Breast Cancer. Mol Cancer Ther.

[133] Wang X, Osada T, Wang Y, Yu L, Sakakura K, Katayama A (2010). CSPG4 protein as a new target for the antibody-based immunotherapy of triple-negative breast cancer. J Natl Cancer Inst.

[135] Wang X, Wang Y, Yu L, Sakakura K, Visus C, Schwab JH (2010). CSPG4 in cancer: multiple roles. Curr Mol Med.

[136] Beard RE, Zheng Z, Lagisetty KH, Burns WR, Tran E, Hewitt SM (2014). Multiple chimeric antigen receptors successfully target chondroitin sulfate proteoglycan 4 in several different cancer histologies and cancer stem cells. J Immunother Cancer.

[137] Harrer DC, Dörrie J, Schaft N (2019). CSPG4 as Target for CAR-T-Cell Therapy of Various Tumor Entities-Merits and Challenges. Int J Mol Sci.

[138] Wei H, Wang Z, Kuang Y, Wu Z, Zhao S, Zhang Z (2020). Intercellular Adhesion Molecule-1 as Target for CAR-T-Cell Therapy of Triple-Negative Breast Cancer. Front Immunol.

[139] Yang L, Froio RM, Sciuto TE, Dvorak AM, Alon R, Luscinskas FW (2005). ICAM-1 regulates neutrophil adhesion and transcellular migration of TNF-alpha-activated vascular endothelium under flow. Blood.

[140] Roland CL, Harken AH, Sarr MG, Barnett CC Jr (2007). ICAM-1 expression determines malignant potential of cancer. Surgery.

[141] Rosette C, Roth RB, Oeth P, Braun A, Kammerer S, Ekblom J (2005). Role of ICAM1 in invasion of human breast cancer cells. Carcinogenesis.

[142] Chaudhary A, Hilton MB, Seaman S, Haines DC, Stevenson S, Lemotte PK (2012). TEM8/ANTXR1 blockade inhibits pathological angiogenesis and potentiates tumoricidal responses against multiple cancer types. Cancer Cell.

[143] Byrd TT, Fousek K, Pignata A, Szot C, Samaha H, Seaman S (2018). TEM8/ANTXR1-Specific CAR T Cells as a Targeted Therapy for Triple-Negative Breast Cancer. Cancer Res.

[144] Bardia A, Mayer IA, Diamond JR, Moroose RL, Isakoff SJ, Starodub AN (2017). Efficacy and Safety of Anti-Trop-2 Antibody Drug Conjugate Sacituzumab Govitecan (IMMU-132) in Heavily Pretreated Patients With Metastatic Triple-Negative Breast Cancer. J Clin Oncol.

[145] Chen H, Wei F, Yin M, Zhao Q, Liu Z, Yu B (2021). CD27 enhances the killing effect of CAR T cells targeting trophoblast cell surface antigen 2 in the treatment of solid tumors. Cancer Immunol Immunother.

[146] Lin H, Zhang H, Wang J, Lu M, Zheng F, Wang C (2014). A novel human Fab antibody for Trop2 inhibits breast cancer growth *in vitro* and *in vivo*. Int J Cancer.

[147] Zagorac I, Lončar B, Dmitrović B, Kralik K, Kovačević A (2020). Correlation of folate receptor alpha expression with clinicopathological parameters and outcome in triple negative breast cancer. Ann Diagn Pathol.

[148] Ginter PS, McIntire PJ, Cui X, Irshaid L, Liu Y, Chen Z (2017). Folate Receptor Alpha Expression Is Associated With Increased Risk of Recurrence in Triple-negative Breast Cancer. Clin Breast Cancer.

[149] Song DG, Ye Q, Poussin M, Chacon JA, Figini M, Powell DJ Jr (2016). Effective adoptive immunotherapy of triple-negative breast cancer by folate receptor-alpha redirected CAR T cells is influenced by surface antigen expression level. J Hematol Oncol.

[150] Lanitis E, Poussin M, Klattenhoff AW, Song D, Sandaltzopoulos R, June CH (2013). Chimeric antigen receptor T Cells with dissociated signaling domains exhibit focused antitumor activity with reduced potential for toxicity *in vivo*. Cancer Immunol Res.

[151] Kim MS, Ma JS, Yun H, Cao Y, Kim JY, Chi V (2015). Redirection of genetically engineered CAR-T cells using bifunctional small molecules. J Am Chem Soc.

[152] Battula VL, Shi Y, Evans KW, Wang RY, Spaeth EL, Jacamo RO (2012). Ganglioside GD2 identifies breast cancer stem cells and promotes tumorigenesis. J Clin Invest.

[153] Cazet A, Bobowski M, Rombouts Y, Lefebvre J, Steenackers A, Popa I (2012). The ganglioside G(D2) induces the constitutive activation of c-Met in MDA-MB-231 breast cancer cells expressing the G(D3) synthase. Glycobiology.

[154] Chen YX, Chen XW, Li CG, Yue LJ, Mai HR, Wen FQ (2013). Effect of tumor gangliosides on tyrosine phosphorylation of p125FAK in platelet adhesion to collagen. Oncol Rep.

[155] Ly S, Anand V, El-Dana F, Nguyen K, Cai Y, Cai S (2021). Anti-GD2 antibody dinutuximab inhibits triple-negative breast tumor growth by targeting GD2(+) breast cancer stem-like cells. J Immunother Cancer.

[156] Nguyen K, Yan Y, Yuan B, Dasgupta A, Sun J, Mu H (2018). ST8SIA1 Regulates Tumor Growth and Metastasis in TNBC by Activating the FAK-AKT-mTOR Signaling Pathway. Mol Cancer Ther.

[157] Seitz CM, Schroeder S, Knopf P, Krahl AC, Hau J, Schleicher S (2020). GD2-targeted chimeric antigen receptor T cells prevent metastasis formation by elimination of breast cancer stem-like cells. Oncoimmunology.

[158] Vivier E, Raulet DH, Moretta A, Caligiuri MA, Zitvogel L, Lanier LL (2011). Innate or adaptive immunity? The example of natural killer cells. Science.

[159] Houchins JP, Yabe T, McSherry C, Bach FH (1991). DNA sequence analysis of NKG2, a family of related cDNA clones encoding type II integral membrane proteins on human natural killer cells. J Exp Med.

[160] Upshaw JL, Arneson LN, Schoon RA, Dick CJ, Billadeau DD, Leibson PJ (2006). NKG2D-mediated signaling requires a DAP10-bound Grb2-Vav1 intermediate and phosphatidylinositol-3-kinase in human natural killer cells. Nat Immunol.

[161] Jin F, Wu Z, Hu X, Zhang J, Gao Z, Han X (2019). The PI3K/Akt/GSK-3β/ROS/eIF2B pathway promotes breast cancer growth and metastasis via suppression of NK cell cytotoxicity and tumor cell susceptibility. Cancer Biol Med.

[162] Groh V, Rhinehart R, Secrist H, Bauer S, Grabstein KH, Spies T (1999). Broad tumor-associated expression and recognition by tumor-derived gamma delta T cells of MICA and MICB. Proc Natl Acad Sci U S A.

[163] Han Y, Xie W, Song DG, Powell DJ Jr (2018). Control of triple-negative breast cancer using *ex vivo* self-enriched, costimulated NKG2D CAR T cells. J Hematol Oncol.

[164] Barber A, Zhang T, Sentman CL (2008). Immunotherapy with chimeric NKG2D receptors leads to long-term tumor-free survival and development of host antitumor immunity in murine ovarian cancer. J Immunol.

[165] Barber A, Zhang T, Megli CJ, Wu J, Meehan KR, Sentman CL (2008). Chimeric NKG2D receptor-expressing T cells as an immunotherapy for multiple myeloma. Exp Hematol.

[166] Parker C (2004). Active surveillance: towards a new paradigm in the management of early prostate cancer. Lancet Oncol.

[167] Li X, Dai D, Chen B, Tang H, Xie X, Wei W (2018). Clinicopathological and Prognostic Significance of Cancer Antigen 15-3 and Carcinoembryonic Antigen in Breast Cancer: A Meta-Analysis including 12,993 Patients. Dis Markers.

[168] Schettini F, Barbao P, Brasó-Maristany F, Galván P, Martínez D, Paré L (2021). Identification of cell surface targets for CAR-T cell therapies and antibody-drug conjugates in breast cancer. ESMO Open.

[169] Ma S, Li X, Wang X, Cheng L, Li Z, Zhang C (2019). Current Progress in CAR-T Cell Therapy for Solid Tumors. Int J Biol Sci.

[170] Zhang BL, Qin DY, Mo ZM, Li Y, Wei W, Wang YS (2016). Hurdles of CAR-T cell-based cancer immunotherapy directed against solid tumors. Sci China Life Sci.

[171] Harlin H, Meng Y, Peterson AC, Zha Y, Tretiakova M, Slingluff C (2009). Chemokine expression in melanoma metastases associated with CD8+ T-cell recruitment. Cancer Res.

[172] Moon EK, Carpenito C, Sun J, Wang LC, Kapoor V, Predina J (2011). Expression of a functional CCR2 receptor enhances tumor localization and tumor eradication by retargeted human T cells expressing a mesothelin-specific chimeric antibody receptor. Clin Cancer Res.

[173] Nishio N, Diaconu I, Liu H, Cerullo V, Caruana I, Hoyos V (2014). Armed oncolytic virus enhances immune functions of chimeric antigen receptor-modified T cells in solid tumors. Cancer Res.

[174] Wang LC, Lo A, Scholler J, Sun J, Majumdar RS, Kapoor V (2014). Targeting fibroblast activation protein in tumor stroma with chimeric antigen receptor T cells can inhibit tumor growth and augment host immunity without severe toxicity. Cancer Immunol Res.

[175] Caruana I, Savoldo B, Hoyos V, Weber G, Liu H, Kim ES (2015). Heparanase promotes tumor infiltration and antitumor activity of CAR-redirected T lymphocytes. Nat Med.

[176] Bollard CM, Rössig C, Calonge MJ, Huls MH, Wagner HJ, Massague J (2002). Adapting a transforming growth factor beta-related tumor protection strategy to enhance antitumor immunity. Blood.

[177] Nishio N, Dotti G (2015). Oncolytic virus expressing RANTES and IL-15 enhances function of CAR-modified T cells in solid tumors. Oncoimmunology.

[178] John LB, Kershaw MH, Darcy PK (2013). Blockade of PD-1 immunosuppression boosts CAR T-cell therapy. Oncoimmunology.

[179] Kakarla S, Chow KK, Mata M, Shaffer DR, Song XT, Wu MF (2013). Antitumor effects of chimeric receptor engineered human T cells directed to tumor stroma. Mol Ther.

[180] Wei X, Lai Y, Li J, Qin L, Xu Y, Zhao R (2017). PSCA and MUC1 in non-small-cell lung cancer as targets of chimeric antigen receptor T cells. Oncoimmunology.

[181] Grupp SA, Kalos M, Barrett D, Aplenc R, Porter DL, Rheingold SR (2013). Chimeric antigen receptor-modified T cells for acute lymphoid leukemia. N Engl J Med.

[182] Sun S, Hao H, Yang G, Zhang Y, Fu Y (2018). Immunotherapy with CAR-Modified T Cells: Toxicities and Overcoming Strategies. J Immunol Res.

[183] Yu S, Yi M, Qin S, Wu K (2019). Next generation chimeric antigen receptor T cells: safety strategies to overcome toxicity. Mol Cancer.

[184] Caruso HG, Hurton LV, Najjar A, Rushworth D, Ang S, Olivares S (2015). Tuning Sensitivity of CAR to EGFR Density Limits Recognition of Normal Tissue While Maintaining Potent Antitumor Activity. Cancer Res.

[185] Fedorov VD, Themeli M, Sadelain M (2013). PD-1- and CTLA-4-based inhibitory chimeric antigen receptors (iCARs) divert off-target immunotherapy responses. Sci Transl Med.

[186] Di Stasi A, Tey SK, Dotti G, Fujita Y, Kennedy-Nasser A, Martinez C (2011). Inducible apoptosis as a safety switch for adoptive cell therapy. N Engl J Med.

[187] Marin V, Cribioli E, Philip B, Tettamanti S, Pizzitola I, Biondi A (2012). Comparison of different suicide-gene strategies for the safety improvement of genetically manipulated T cells. Hum Gene Ther Methods.

[188] Tiberghien P, Ferrand C, Lioure B, Milpied N, Angonin R, Deconinck E (2001). Administration of herpes simplex-thymidine kinase-expressing donor T cells with a T-cell-depleted allogeneic marrow graft. Blood.

